# The impact of the use of new technologies on farmers’ wheat yield in Ethiopia: evidence from a randomized control trial

**DOI:** 10.1111/agec.12425

**Published:** 2018-07-02

**Authors:** Gashaw Tadesse Abate, Tanguy Bernard, Alan de Brauw, Nicholas Minot

**Affiliations:** ^1^ International Food Policy Research Institute Addis Ababa Ethiopia; ^2^ International Food Policy Research Institute Washington DC 20005; ^3^ GREThA University of Bordeaux Pessac France 33600

**Keywords:** O11, O13, Q12, Agricultural practices, Yield, Randomized controlled trial, Ethiopia

## Abstract

In 2013, Ethiopia's Agricultural Transformation Agency introduced the Wheat Initiative to increase smallholder productivity. In this article, we measure the impacts of the Wheat Initiative package of technologies, and its marketing assistance component alone, on yields among a promotional group of farmers. The package includes improved techniques, improved inputs, and a guaranteed market for the crop. Relying on crop‐cut measures and farmers’ own assessments, we find that full package led to an average 14% higher yields. Implementation of the Wheat Initiative was successful in making certified seed and fertilizer accessible to farmers and increasing their uptake, though only 61% of the intervention group adopted row planting and few farmers received marketing assistance. The measured yield difference may underestimate the true yield difference associated with the technology because of incomplete adoption of the recommended practices by intervention farmers and adoption of some practices by control farmers.

## 1. Introduction

Yields are often much lower than their potential among smallholder farmers in sub‐Saharan Africa (World Bank, 2007). Smallholders may not adopt modern inputs or farming techniques that would increase their productivity because they face one of several constraints (e.g., Jack, 2011; Shiferaw et al., 2015). Meanwhile, governments in sub‐Saharan Africa have renewed efforts to increase local food production by promoting the use of modern inputs and practices (Jayne and Rashid, 2013; Rashid et al., 2013). Large‐scale programs supporting smallholder use of improved seeds and chemical fertilizers have begun, such as Malawi's fertilizer subsidy program (Dorward and Chirwa, 2011). Such programs function on the premise that with better modern input availability, smallholder farmers can close the gap between their productivity levels and levels found in local experiment stations.

Meanwhile, a body of research is emerging that experiments with methods of alleviating constraints on smallholder farmer productivity. Many interventions attempt to relax one or two key constraints on productivity, which include input availability, liquidity, risk, information, and poorly functioning output markets (Jack, 2011). For example, Duflo et al. (2011) test a program in which farmers could buy fertilizer with free delivery right at harvest in Kenya, alleviating liquidity and input availability constraints, and find positive impacts on profitability. Carter et al. (2013) find that discount vouchers have similar positive impacts on improved seed and fertilizer adoption in Mozambique. Similarly, Liverpool‐Tasie (2014) shows that using vouchers to distribute subsidies increases farmers access to fertilizer in Nigeria. In Malawi, Beaman et al. (2015) show that farmers need several information sources to relax risk and information constraints and induce adoption of new seeds. Also in Malawi, BenYishay and Mobarak (2017) test various methods of using social networks to ease information constraints.

Other authors have attempted to relax liquidity and risk constraints, sometimes in the same projects. Ambler et al. (2017) find large positive impacts on production from relaxing liquidity constraints on farmers in Senegal. Karlan et al. (2014) improve liquidity with cash grants and reduce risk with insurance among farmers in northern Ghana, finding larger impacts from insurance than from grants. Beaman et al. (2014) test various methods of relaxing liquidity constraints in Mali (cash grants versus credit) and find larger impacts from grants. In line with reducing uninsured risks, a recent study in Ethiopia by Alem and Boroussard (2018) find that participation in a food‐for‐work program increases fertilizer adoption by allowing farmers to take on more risks.

From the perspective of information and markets, Bernard et al. (2017) introduce third‐party quality certification in local onion markets and find positive impacts on prices received by Senegalese farmers and on their use of quality‐enhancing fertilizers. Also, related to poorly functioning output markets, Mitra et al. (2018) attempt to improve potato farmers’ bargaining position with middlemen through price information and find that a lack of competition among buyers completely constrains the pass through of price changes in higher level markets to farmers.

All these papers share two particularly salient features. First, they all attempt to relax one or two constraints on agricultural productivity. Relaxing these constraints should lead to farm‐level reoptimization of production decisions. But if farmers still face other constraints, their outcomes will remain suboptimal (Foster and Rosenzweig, 2010). Moreover, so long as market access and farmer ability are heterogeneous, the profitability of adopting modern inputs or techniques will be heterogeneous (Suri, 2011). Second, if government agencies put these approaches in place on a large scale, they may not achieve the same results. Unlike small interventions managed and monitored by NGOs, government interventions need large quantities of inputs of homogenous quality delivered on time to farmers in sometimes remote locations, functional and well‐targeted extension services, and a level of trust in their services by farmers. In Kenya, Bold et al. (2016) compare results from similar educational interventions implemented by NGOs and the government and find children's learning outcomes differ substantially. The same principle may hold for the agricultural sector.

In this article, we add to the literature by evaluating the introduction of the Wheat Initiative of Ethiopia's Agricultural Transformation Agency (ATA). The Wheat Initiative includes the use of improved inputs, improved farming techniques, and a guaranteed market for output, thus simultaneously addressing multiple constraints faced by smallholders. Material inputs in the package include certified wheat seed, urea, and diammonium phosphate (DAP) as fertilizers, and gypsum to improve soil structure. Improved techniques in the package include lower seeding rates, row planting, and balanced fertilizer use. Third, before the planting season began, the government committed to buy farmers’ wheat at the market price or above, to reduce marketing risk for farmers. The evaluation measures outcomes among randomly selected promotional farmers selected within each kebele. The promotional farmers received training on agronomic practices, certified wheat seed on credit, urea fertilizer, and gypsum as an in‐kind per diem for participating in agronomic practice training, and marketing assistance after harvest.^1^ A second treatment group only received marketing assistance. Through individual‐level randomization, we can present impact estimates controlling for unobservable factors at the kebele level.

The government expected that farmers would adopt the entire package, which would approximately double yields. But the impact of a package on yields among smallholders is largely unknown, in part because the extent to which farmers will adopt package components is unclear. Although implementing a package intervention does not allow us to disentangle the impacts of specific package components, in this article our primary goal is to measure average impacts of the Wheat Initiative among promotional farmers and to examine package components that farmers took up.^2^

We find the complete wheat package increased yields by approximately 14% at harvest. While sizeable, the increase is well below the ATA's initial expectations of doubled yields based on agronomic trials (Abraham et al., 2014). Absent input support, we find the marketing assistance intervention alone did not affect yields. Many program components were well executed; for example, inputs were made accessible on time, uptake was high, beneficiaries were satisfied with the quality of inputs purchased or received, and extension services reached all targeted farmers and effectively raised knowledge. Only the marketing support component had significant issues: only 16% of targeted farmers reporting receiving marketing assistance. The poor implementation of the market component suggests farmers may not have found the purchase guarantee credible. We further find that farmers partially implement recommendations of the wheat package. The seeding rate among beneficiaries is lower than among control households but remains 53% higher than recommended. Similarly, treated farmers use 26% less fertilizer than recommended on average, and only 61% of treated farmers used row planting.

A second contribution is that we use three different measures of yields: a crop cut, in which a small area of the field was harvested, and the output weighed; a measure that uses a farmer report of wheat production, but the actual field area measured with the polygon method; and a measure that uses farmer reports of wheat production and sown area. Not surprisingly, results differ somewhat by method, which has implications for measurement of impact estimates. While we find consistent estimates between crop‐cut measures and farmers’ preharvest expectation of yields, postharvest recall data show a lower impact of the intervention that is not statistically significant—though point estimates are higher than with the crop‐cut —suggesting potential non‐classical measurement errors in such measures.

## 2. Context

### 2.1. Wheat production in Ethiopia

Wheat is typically grown by smallholders in Ethiopia's highlands and is an important component of Ethiopia's production system. Recent estimates show that wheat farmers in Ethiopia produce, on average, 2.1 t/ha, well below the experimental yield of above 5 t/ha (Hailu, 1991; MOA, 2012). Yields in Ethiopia consistently lag behind average yields in Africa and beyond. In 2012, for instance, Ethiopia's wheat yields were on average 29% below neighboring Kenya, 13% below the African average, and 32% below the world average (FAO, 2014).

Several socioeconomic, abiotic, and biotic constraints combine to explain the gap between actual and potential yields. First, wheat farmers are not likely to use modern production‐enhancing inputs, such as certified seeds and fertilizers.^3^ The 2012 national estimates on input use indicate that only 8.4% of wheat area was planted with certified seeds and 48% was fertilized. When fertilizer is applied, estimated average application rate is 48 kg/ha, well below the recommended rate of 200 kg/ha (Endale, 2010; MOA, 2012; Spielman et al., 2011).

Abiotic factors, such as low and poor distribution of rainfall in lowland areas, plant lodging in half of the highlands, soil erosion, disease, and weeds also contribute to significant wheat yield losses in the country. For instance, Zewdie et al. (1990) suggest that plant lodging causes yield losses of 10–30%. Estimated yield gains with proper weed control (through row planting and reduced seeding rates) range from 35–85% (Bogale et al., 2011; Desta, 2000; Tessema et al., 1996; Tessema and Tanner, 1997).

Finally, Ethiopian farmers traditionally plant wheat seeds using hand broadcasting. Compared with direct seeding, broadcasting reduces yields due to poorer seed‐to‐soil contact and delayed germination, higher competition between plants for inputs because of uneven seed distribution, and difficulty in controlling grassy weeds. While row planting can limit these issues, its higher labor requirement undermines economic viability (Moser and Barrett, 2006; Vandercasteelen et al., 2013).

The results of on‐station and on‐farm trials following the System of Wheat Intensification have led to optimism about the potential of comprehensive extension package to address these constraints and increase wheat productivity (Abraham et al., 2014).^4^ For instance, on‐farm trials in northern Ethiopia and South Wollo showed that optimal use of inputs, row planting with reduced seeding rate, and proper implementation of agronomic best practices increased wheat yield by an average factor of 2.7 relative to control plots (4.9 t/ha vs. 1.8 t/ha).

### 2.2. The ATA Wheat Initiative

**Figure 1:**
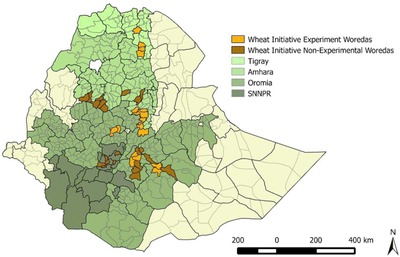
Map of the Wheat Initiative regions and *woredas*. *Source*: Authors. [Color figure can be viewed at http://wileyonlinelibrary.com]

To simultaneously address multiple constraints on wheat productivity, Ethiopia's Ministry of Agriculture, with the support of the Ethiopian ATA, launched the Wheat Initiative in the 2013 *meher* rainy season. The Wheat Initiative rolled out a promotional package, including training, inputs, and the market component, for 2,000 “benchmark” farmers in 41 *woredas* in the four major wheat‐producing regions (Fig. 1). Benchmark farmers were required to have a wheat plot of at least 0.5 ha and to be open to changes in agricultural practices. Selected farmers originated from 200 *kebeles* within the 41 *woredas* and were typically made up of two model farmers, two nonmodel farmers, and one female farmer.^5^ The wheat package was meant to be implemented on benchmark farmers’ 0.5‐ha plots. First, farmers received one day of training, including messages regarding row planting, fertilizer recommendations and a lower seeding rate. Second, farmers received 50 kg of certified improved seed on credit (free of interest) and 50 kg of urea fertilizer and 25 kg of gypsum for free; the justification was that the inputs were an in‐kind payment for training participation. Third, benchmark farmers were informed that the Ethiopian Grain Trade Enterprise (EGTE) would purchase their wheat at or above the prevailing market price.^6^

## 3. Study design

### 3.1. Sample and allocation of treatment

Our study relies on 36 experimental *kebeles* spanning 18 *woredas* in the Oromia, Amhara, and Tigray regions (Fig. 1). While the study randomly selected *woredas* for the study in Oromia (7 out of 15 *woredas*) and Amhara (7 out of 16 *woredas*) regions, it includes all four targeted *woredas* in Tigray, since the intervention targeted fewer *woredas* with comparable production potential in Tigray. The study did not include intervention *woredas* in the SNNP region, as implementation occurred there earlier. The sample design followed a three‐stage approach. First, 18 *woredas* as above were asked to send a list of 14 farmers per *kebele*, including at least 4 model farmers and 2 female farmers.^7^ Second, two *kebeles* per *woreda* were randomly selected for the intervention.

Third, within each *kebele* the 14 farmers were randomized into benchmark farmers, market farmers, and control farmers (504 wheat farmers in total). To ensure sample *kebeles* maintained the mix of model, nonmodel, and female farmers targeted by the program, randomization within each *kebele* was stratified to ensure two model farmers, two nonmodel farmers, and one female farmer were selected for the full‐package group. The evaluation design therefore compares yield outcomes among benchmark farmers who receive the full package as above, a group of farmers who receive only the EGTE market guarantee, and a control group. Note that farmers in the control group were neither restricted from purchasing improved inputs without subsidies nor prevented from learning about the techniques on their own.

### 3.2. Data

The article uses two primary data sources. First, it uses a crop‐cut measurement survey, which was conducted in November and December 2013 in all 36 *kebeles*. This survey aimed at measuring wheat outputs and plot areas for all three groups of farmers, just before harvest. Each plot was measured output in two ways: a sample crop‐cut output measurement (wet weight) and farmers’ preharvest estimates of wheat outputs from the whole plot. Experts from the Central Statistical Agency (CSA) trained in measuring wheat yields carried out the crop‐cut. Of the 504 experimental and control farmers, wheat production was successfully measured on 382 plots.^8^ Second, a wheat growers’ survey was conducted in February and March 2014, after harvest was complete. The survey covered 490 farmers from all three groups, and gathered information related to input use, labor use, land use, wheat production and marketing. It also asked questions about farmers’ social networks and plans for growing wheat using the wheat package in the following season.

### 3.3. Yield measurement

Our primary outcome of interest is wheat yield, defined as the amount of harvested wheat output divided by plot area, a preferred denominator to harvested area since the latter overlooks possible losses in crop area between planting and harvesting (Reynolds et al., 2015). Estimating the average wheat yield on a specific plot requires estimating both the plot area and the quantity of output obtained, which are challenging to measure and prone to errors in this context. For example, local units used for measuring quantities and areas are inconsistent from one location to another. Thus, the evaluation used alternative estimates of crop area and harvested product to ensure the robustness of yield estimates.

**Figure 2:**
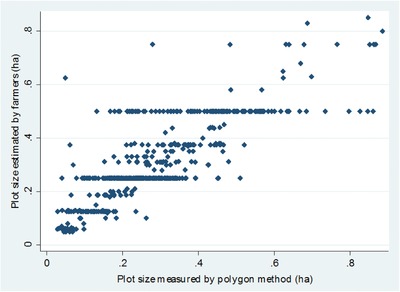
Scatter plot between plot size measured by polygon method and farmer assessment. *Source*: Authors’ calculation based on crop‐cut survey data. [Color figure can be viewed at http://wileyonlinelibrary.com]

By design, wheat plots among selected farmers should have had an area of 0.5 ha. However, during field visits, significant deviations from the standardized area were found. Thus, during crop‐cut and wheat growers’ surveys, both experimental and control plots were subject to area measurement. The plot area was measured using the polygon method (direct area measurement) and through self‐reports. The polygon method involves the use of a rope and a compass to measure the length of each side and the angle of each corner; this information was then used to calculate the plot size. Local units were used for self‐reports of plot area, to reduce potential rounding errors while converting to standard units. While there is a correlation of 0.76 between the polygon measure and self‐reports, like Carletto et al. (2015) we find that farmers often round off their estimated plot size, represented by horizontal lines (Fig. 2).

We use three different measures of wheat output. First, the crop cut occurred just before harvest on a random 4 × 4 m subplot of the wheat plots. The output from this random subplot was harvested and threshed, and enumerators measured the wet weight. Second, at the time of the crop‐cut exercise, farmers were asked to predict their expected output from the entire plot. The prediction was made in the presence of the enumerator and within sight of the plot, so enumerators could judge the validity of farmers’ estimates. Third, postharvest estimation of output was obtained from farmers during the wheat growers’ survey, which was conducted after harvesting, drying, and threshing were completed.

Each yield measure has strengths and weaknesses. Yields from crop‐cut measurement are often considered the most reliable and objective measure, but they do not account for postharvest losses during drying, threshing, cleaning, and transportation. Moreover, the process of locating a subplot for crop‐cut can be subject to selection bias (such as excluding the plot border). Finally, conducting a crop‐cut is logistically challenging due to spatial variation on the harvest calendar. Crop‐cut data could not be collected from 20% of sampled farmers who have higher yields than other farmers based on the expected yield measure (Supporting Information Appendix Table A.1). The benefit of yield estimates based on farmers’ preharvest predictions is that it better reflects the attainable yield (Fermont and Benson, 2011; Reynolds et al., 2015), though it may be subject to social desirability bias. Finally, farmer recall reflects the product perceived useful to the farmer but is subject to rounding biases in both numerator and denominator. Overall, yield measurements based on farmer prediction and recall allows the collection of a larger set of yield estimates than crop‐cuts alone.

## 4. Empirical strategy

### 4.1. Experimental integrity

First, we examine primary household characteristics that should be relatively time invariant, by treatment group (Table 1). A Wald test of the equality of means across all three groups was used, with the results shown in the last column. We find significant differences in gender of household head as well as household size. Female farmers make up a larger share of the full‐package group (20%) than either of the other two groups (11% and 9% in marketing‐only and control groups, respectively). The imbalance on gender is due to limited number of female farmers in the experimental sample—that is, two female farmers per kebele were randomized for three study groups. Farmers in the control group also tended to have somewhat larger households (7.2 members) compared to the other two groups (6.7 and 6.4 members in full‐package and marketing‐only groups, respectively).

**Table 1:** Characteristics of households in each treatment group

Variable	Full‐package farmers (*n* = 197)	Marketing farmers (*n* = 126)	Control farmers (*n* = 167)	*F*‐test of differences in means
HH head age (year)	45.5	44.0	47.1	2.87*
HH head gender (1 = male, 0 = female)	0.79	0.89	0.91	11.84***
HH head education (in completed years)	2.62	2.63	2.56	0.23
Household size (number)	6.69	6.44	7.20	8.34***
Landholding size (ha)	2.37	2.22	2.30	0.28
Irrigated land size (ha)	0.015	0.018	0.039	0.99
Red‐colored soil (1 = yes)	0.218	0.246	0.311	3.10*
Black‐colored soil (1 = yes)	0.594	0.540	0.474	3.19*
Gray/sand‐colored soil (1 = yes)	0.188	0.214	0.216	0.33
Distance to wheat plot (minutes)	13.7	15.4	14.3	0.55
Radio ownership (1 = yes)	0.73	0.71	0.69	0.33
Television ownership (1 = yes)	0.14	0.18	0.11	0.89
Cellphone ownership (1 = yes)	0.83	0.73	0.72	4.69**
Bicycle ownership (1 = yes)	0.02	0.03	0.02	0.20
Car ownership (1 = yes)	0.03	0.04	0.02	0.42
Livestock ownership (number, TLU)	11.8	10.4	10.8	2.14
Housing (number of distinct units)	2.68	2.71	2.69	0.04
Agricultural tools owned (number)				
Axe	2.37	2.25	2.41	0.81
Pick‐axe	1.71	1.76	1.60	0.52
Sickle	3.99	4.06	4.04	0.05
Plough	2.17	2.09	2.08	0.28
Yoke	1.99	1.94	1.94	0.20
Hay fork	2.26	2.12	2.03	2.37
Shovel	1.19	1.25	1.14	0.72
Hoe	1.71	1.65	1.47	1.50
Winnower	1.47	1.46	1.39	0.74
Cart	0.22	0.23	0.22	0.00
Water pump	0.06	0.03	0.08	0.91

*Source*: Authors’ calculation based on the data from the crop‐cutting exercise and the 2014 wheat growers’ survey.

*Notes*: Number of observation = 490. Statistically significant difference with the control farmers *** at the1% level, ** at the 5% level, and * at the 10% level.

We also uncover evidence of differences in the age of the farmer, ownership of cellphone, and the share of red and black soil. Control farmers tended to be somewhat older than those in the other groups, with the difference being two to three years, on average. Control farmers were also more likely to have red soils and less likely to have black soils compared to the other two groups. On the other hand, the vast majority of farmers in the full‐package group tended to have cellphones (83%) compared to the farmers in market‐only (73%) and control groups (72%). There were no statistically significant differences in education, housing, asset ownership (e.g., land, livestock, radio, television, and car) agricultural tools (e.g., plough, yoke, sickle, hay fork, winnower, and cart), the share of gray/sandy soils, and distance to plot. Given these slight imbalances across treatment groups, we include all variables in Table 1 in estimating impacts.

### 4.2. Estimation strategy

We estimate the effect of the full wheat package and the market‐only aspect of the package based on Eq. (1):

(1) $$\ln Y_{ik}=\alpha_{k}\;+\beta T_{ik}+\gamma G_{ik}+u_{ik},$$

where *Y* represents yields; *T* represents the full‐package group, the primary treatment; *G* represents the marketing‐only group; and uis a mean‐zero error term. The index *i* represents households, and *k* indexes *kebeles*, so α*_k_* indicates the use of *kebele‐*level fixed effects. The primary null hypotheses to be tested are whether *β* (the difference in yields between full‐package farmers and control farmers) and γ(the difference in yields between marketing‐only farmers and control farmers) are zero.

For our main estimates, we make three additions to the model. First, we add indicator variables for model farmers *M*, and female farmers *F*; we add interaction terms with the primary treatment group to examine heterogeneous treatment effects for model farmers and female farmers; and we control for a vector of household and plot characteristics *Z*, reflecting variables from Table 1, that are unlikely to have changed over time. The resulting specification is

(2) $$\begin{array}{ccc} \ln Y_{ik} & = & \alpha_{k}\;+\beta T_{ik}+\theta_{1}T_{ik}M_{ik}+\theta_{2}T_{ik}F_{ik}+\gamma G_{ik}+\delta_{1}M_{ik}\\ & & +\;\delta_{2}F_{ik}+Z_{ik}^{\prime }\omega +u_{ik}. \end{array}$$

## 5. Results and discussion

### 5.1. Descriptive evidence

Table 2 presents naive differences in wheat yield across treatment groups for the three yield measures. Yields are higher among benchmark farmers than the other two groups regardless of measurement method. According to the crop‐cut, yields averaged 2.92 t/ha among the full‐package farmers, compared with 2.77 and 2.73 t/ha among the market and control farmers, respectively. Although estimates among all three groups are higher than the average yields in Ethiopia as a whole (2.1 t/ha), even among the full‐package farmers they are well below the average for treatment plots in agronomic trials (4.9 t/ha). Yield estimates from farmers’ predicted output preharvest and farmers’ recalled output postharvest are higher on average compared to the crop‐cut, which partially reflects that farmers who harvested early had higher yields.

**Table 2:** Area, production (output), and yield estimates

Variable	Full‐package farmers (*n* = 197)		Marketing farmers (*n* = 126)		Control farmers (*n* = 167)	
	Mean	SD	Mean	SD	Mean	SD
Plot size (ha)						
Measured	0.30	0.01	0.37	0.04	0.45	0.06
Estimated by farmers	0.33	0.01	0.43	0.05	0.45	0.05
Output (production)						
Crop‐cut (4 × 4 m^2^) (kg)	4.68	0.41	4.43	0.32	4.37	0.33
Farmer prediction (*t*)	1.19	0.11	1.27	0.19	1.55	0.26
Farmer recall (*t*)	1.98	0.24	2.25	0.24	2.11	0.25
Yield estimates (*t*/ha)						
Yield based on crop‐cut	2.92	0.25	2.77	0.20	2.73	0.21
Yield based on farmer prediction	3.69*	0.20	3.26	0.18	3.22	0.22
Yield based on farmer recall	3.18	0.23	3.07	0.20	2.92	0.21

*Source*: Authors’ calculations based on the crop‐cutting exercise and the 2014 wheat growers’ survey. Crop‐cut average is actual output harvested from each subplot (in kilograms).

*Statistically significant difference with the control farmers at the 10% level.

### 5.2. Impact estimates

In the following tables, we report the intent‐to‐treat estimates of the wheat package and marketing assistance only on farmers’ yields using the three yield measures. First, we use the crop‐cut (Table 3). With only the two treatment variables in the regression, we find a positive coefficient on the full‐package indicator (0.079), but it is not significantly different from zero (column 1). When we control for farmer types, the coefficient estimate increases to 0.095, but remains statistically insignificant (column 2). We then sequentially include interaction terms between the full‐package treatment and model and female farmer indicators and household‐ and plot‐level control variables (columns 3 and 4). The inclusion of these variables increases the full‐package coefficient to 0.124 and 0.140, respectively, and the latter estimate is significant at the 5% level. The last coefficient implies an increase in yields of about 15% for farmers receiving the full package relative to the control group. Although these results may not appear to be strong evidence of an impact of the Wheat Initiative, note that the sample size was reduced as some farmers had already harvested at the time of the crop‐cut, so statistical power is not as high as in the full sample.^9^ Coefficients on the marketing assistance indicator and the interaction terms are not significantly different from zero; the former implies that the marketing guarantee did not affect yields, while the latter suggests that either impacts across farmer types are not statistically different or the sample does not have the statistical power to detect differences.

**Table 3:** The impact of the promotional wheat package on farmers’ wheat yield based on crop‐cut estimates

Explanatory variable	Dependent variable: yield based on crop‐cut			
	(1)	(2)	(3)	(4)
Full package	0.079	0.095	0.124*	0.140**
	(0.058)	(0.060)	(0.070)	(0.070)
Marketing assistance	−0.020	−0.017	−0.014	−0.004
	(0.066)	(0.066)	(0.066)	(0.066)
Model farmer		−0.021	0.048	0.044
		(0.058)	(0.081)	(0.084)
Female farmer		−0.099	−0.190*	−0.144
		(0.073)	(0.113)	(0.114)
Treatment × model			−0.150	−0.121
			(0.116)	(0.120)
Treatment × female			0.146	0.113
			(0.154)	(0.154)
Age of household head				−0.004
				(0.003)
Education of household head				0.017
				(0.030)
Landholding size				0.026
				(0.020)
Household size				0.005
				(0.012)
Black soil				−0.222***
				(0.069)
Gray/sandy soil				−0.050
				(0.067)
Distance to plot				<0.001
				(0.002)
Radio ownership				−0.079
				(0.062)
Television ownership				−0.108
				(0.093)
Cellphone ownership				−0.003
				(0.067)
Bicycle ownership				−0.107
				(0.119)
Car ownership				0.068
				(0.156)
Livestock ownership (in TLU)				<0.001
				(0.005)
Kebele fixed effect	Yes	Yes	Yes	Yes
Observations	367	367	367	367
*R* ^2^	0.514	0.516	0.522	0.546

*Source*: Authors’ calculations based on the crop‐cut and the 2014 wheat growers’ survey.

*Notes*: Robust standard errors in parentheses are calculated at the kebele level. Statistically significant difference *** at the 1% level, ** at the 5% level, and * at the 10% level. In the interaction terms, *treatment* refers to the full‐package treatment.

When farmers’ preharvest prediction of output and actual plot size are used to compute yields, results are broadly consistent (Table 4). Average impacts among the full‐package group are always significant at the 10% level, and coefficient estimates in columns 3 and 4 (0.135) are significant at the 5% level. The coefficient estimate implies that the full package led to an increase in wheat yields of 14.4%. As in Table 3, the yield impact estimates indicate no significant impact among those farmers only receiving marketing assistance. The results in Table 4 also suggest that female benchmark farmers did not do as well; the coefficient on the interaction between the full‐package and female farmers is negative and significant, suggesting that the female farmers gained less than male farmers from the full package.

**Table 4:** The impact of the promotional wheat package on farmers’ wheat yield based on farmer prediction of output

Explanatory variable	Dependent variable: yield based on crop‐cut			
	(1)	(2)	(3)	(4)
Full package	0.102*	0.102*	0.135**	0.135**
	(0.052)	(0.053)	(0.057)	(0.059)
Marketing assistance	0.004	0.003	−0.001	−0.001
	(0.061)	(0.061)	(0.061)	(0.062)
Model farmer		0.047	0.035	0.031
		(0.050)	(0.068)	(0.070)
Female farmer		−0.047	0.122	0.172
		(0.084)	(0.136)	(0.126)
Treatment × model			0.019	<0.001
			(0.100)	(0.104)
Treatment × female			−0.293*	−0.299*
			(0.173)	(0.167)
Age of household head				0.001
				(0.003)
Education of household head				0.038
				(0.035)
Landholding size				−0.003
				(0.018)
Household size				0.004
				(0.012)
Black soil				−0.098
				(0.071)
Gray/sandy soil				−0.146*
				(0.081)
Distance to plot				−0.000
				(0.002)
Radio ownership				−0.062
				(0.059)
Television ownership				0.090
				(0.066)
Cellphone ownership				0.134*
				(0.071)
Bicycle ownership				0.116
				(0.115)
Car ownership				0.015
				(0.137)
Livestock ownership (in TLU)				<0.001
				(0.004)
Kebele fixed effect	Yes	Yes	Yes	Yes
Observations	482	482	482	482
*R* ^2^	0.361	0.363	0.370	0.389

*Source*: Authors’ calculations based on the crop‐cut and the 2014 wheat growers’ survey.

*Notes*: Robust standard errors in parentheses are calculated at the kebele level. Indicates statistically significant difference *** at the 1% level, ** at the 5% level, and * at the 10% level. In the interaction terms, *treatment* refers to the full‐package treatment.

Third, we estimate impacts using yields computed based on farmers’ recall of output after harvest completion combined with farmer assessment of plot size (Table 5). Contrasting with previous estimates, we find no statistically significant relationship between the full‐package indicator variable and wheat yields, regardless of specification. Recall that measurement error in the denominator will cause nonclassical measurement error in the outcome; these results suggest caution to researchers looking to use farmer self‐reports of sown area to compute yields. Overall, the 14% increase in yields due to the Wheat Initiative is relatively substantial, since treatment farmers were encouraged to simply use existing technologies. While well below yields from experimental stations, the estimated wheat yield gain is similar to the impact of teff row planting, part of the teff technology package promoted by MoA with the support of the ATA in Ethiopia, of between 2% and 12% (Vandercasteelen et al., 2013).

**Table 5:** The impact of the promotional wheat package on farmers’ wheat yield based on farmer recall of output

Explanatory variable	Dependent variable: yield based on crop‐cut			
	(1)	(2)	(3)	(4)
Full package	0.051	0.034	0.044	0.039
	(0.056)	(0.057)	(0.066)	(0.068)
Marketing assistance	0.026	0.023	0.023	0.005
	(0.065)	(0.065)	(0.065)	(0.066)
Model farmer		0.129**	0.166**	0.188***
		(0.052)	(0.069)	(0.071)
Female farmer		−0.022	−0.091	−0.081
		(0.079)	(0.135)	(0.134)
Treatment × model			−0.078	−0.109
			(0.108)	(0.114)
Treatment × female			0.113	0.118
			(0.163)	(0.163)
Age of household head				−0.002
				(0.003)
Education of household head				0.011
				(0.029)
Landholding size				−0.032
				(0.023)
Household size				−0.013
				(0.013)
Black soil				−0.016
				(0.081)
Gray/sandy soil				0.121
				(0.082)
Distance to plot				−0.002
				(0.002)
Radio ownership				−0.054
				(0.064)
Television ownership				0.166**
				(0.076)
Cellphone ownership				−0.055
				(0.073)
Bicycle ownership				−0.040
				(0.118)
Car ownership				−0.226*
				(0.122)
Livestock ownership (in TLU)				0.010*
				(0.006)
Kebele fixed effect	Yes	Yes	Yes	Yes
Observations	489	489	489	489
*R* ^2^	0.449	0.456	0.458	0.478

*Source*: Authors’ calculations based on the crop‐cut and the 2014 wheat growers’ survey.

*Notes*: Robust standard errors in parentheses are calculated at the kebele level. Indicates statistically significant difference *** at the 1% level, ** at the 5% level, and * at the 10% level. In the interaction terms, *treatment* refers to the full‐package treatment.

### 5.3. Pathways to impact

Given the results from the first two yield measurements, we next explore pathways that could have led to these impacts using a set of questions related to farmers’ knowledge of the wheat package components, their experience in receiving intervention components, and whether they implemented recommendations. Note that for control‐group farmers, receipt of a package input implies they purchased it. Farmers were also asked for opinions about Wheat Initiative recommendations and plans for implementing recommendations for the next year, both of which are imperfect indicators of the likelihood that farmers will sustain adoption in the future.

#### 5.3.1. Knowledge

Table 6 first investigates differences in knowledge across the study groups. Almost all the full‐package benchmark farmers were aware of the Wheat Initiative, whereas just over half of the marketing‐only and control‐group farmers knew of it. There is no evidence that the marketing‐only farmers knew the package better than the control group. There is also a large, statistically significant difference between farmers in the full‐package group and in the other two groups in terms of their access to wheat production method trainings (59.1 percentage points; row 2), although we find that the marketing group reports receiving training on wheat production methods 10 percentage points more often than the control group. Model farmers are also more likely to have participated in trainings than regular farmers.

**Table 6:** Farmers’ knowledge of the promotional wheat package

Outcome	Information on ATA Wheat Initiative (%, yes)	Training on wheat production method (%, yes)	Package include certified seed (%, yes)	Package include reduced seed rate (%, yes)	Package include row planting (%, yes)	Urea application rate (kg/ha)	DAP application rate (kg/ha)
Full package	0.392***	0.593***	0.011	0.060	−0.003	−1.706	−4.671
	(0.050)	(0.051)	(0.017)	(0.044)	(0.040)	(8.089)	(8.924)
Marketing assistance	−0.024	0.103*	0.022	−0.024	−0.065*	3.562	−2.126
	(0.059)	(0.055)	(0.016)	(0.046)	(0.037)	(6.951)	(7.097)
Model farmer	0.065	0.227***	0.016*	0.067	0.051	−5.814	−5.127
	(0.068)	(0.067)	(0.009)	(0.049)	(0.035)	(9.838)	(8.058)
Female farmer	−0.004	0.029	−0.078	−0.055	−0.065	2.200	1.908
	(0.099)	(0.086)	(0.058)	(0.098)	(0.085)	(8.295)	(9.179)
Treatment × model	−0.121	−0.169**	−0.004	−0.024	−0.034	18.627	5.463
	(0.084)	(0.080)	(0.014)	(0.066)	(0.059)	(11.976)	(11.525)
Treatment × female	0.019	−0.063	0.088	0.017	0.071	0.780	5.261
	(0.111)	(0.101)	(0.058)	(0.115)	(0.099)	(11.140)	(13.069)
Other control variables?	Yes	Yes	Yes	Yes	Yes	Yes	Yes
Kebele fixed effect	Yes	Yes	Yes	Yes	Yes	Yes	Yes
Control group mean	57.4	38.3	94.4	87.9	91.1	163.5	185.3
Observations	490	490	472	490	490	430	432
*R* ^2^	0.256	0.417	0.109	0.186	0.411	0.545	0.583

*Notes*: Authors’ calculations based on the 2014 wheat growers’ survey. Robust standard errors in parentheses. ***P < 0.01, **P < 0.05, *P < 0.1. Full results available in Supporting Information Appendix Table A.3.

Full‐package farmers are much more likely to be trained or have heard of the Wheat Initiative, but they do not appear to know more of the Wheat Initiative recommendations than the other groups (rows 3–7). More than 90% of farmers in all three groups heard about the new wheat recommendations or package from other sources, and almost all respondents knew they should use certified seed. Farmers generally also knew about the recommendations to reduce the seeding rate (85–91%) and use row planting (83–91%). There were differences in farmers’ knowledge of recommended fertilizer application rates, though all three groups of farmers overestimated recommended fertilizer application rates on average. Full‐package farmers suggested lower recommended rates than other farmers, indicating that their estimates were closer to the actual recommendation rates. Overall, while we find some differences on access to information and training, we do not uncover large differences in farmers’ knowledge regarding production practices. This finding implies either initially high knowledge of such practices or large knowledge spillovers from full‐package farmers to control‐group farmers.

#### 5.3.2. Inputs used

We next report farmers’ experiences with the services provided by the Wheat Initiative by treatment status (Table 7). As noted earlier, full‐package farmers were supposed to receive three major incentives to participate in the Wheat Initiative: (1) certified seeds on credit, (2) urea and gypsum for free, and (3) training on agronomic best practices, mainly on row planting and reduced seeding rate. We find that most full‐package farmers received certified seed on time; the treatment effect is 66.3 percentage points (column 1). However, 29.3% of control group farmers reported purchasing or receiving certified seed, suggesting there was measurable “leakage” into the control group. A significant percentage of full‐package farmers indicated that they received the urea and gypsum on time, relative to the control group (columns 2 and 3). However, we find control‐group farmers who received inputs that were supposed to be given only to the full‐package farmers that attend the agronomic training as in‐kind per diem—16.7% of the control group reported receiving urea and 4.7% reported receiving gypsum. That said, a larger share of full‐package farmers who received seed reported it was of very good quality (as subjectively measured by the extent of damage, foreign matter, uniform size and variety, and germination rate) than in the market assistance or control groups; virtually all full‐package farmers reported very good quality seed versus 65.3% of the control group.

**Table 7:** Farmers’ experiences with the services provided under the Wheat Initiative

Variables	Received certified seed (%, yes on time)	Received Urea for free (%, yes on time)	Received gypsum for free (%, yes on time)	Received marketing assistance (%, yes)	Grow wheat differently in 2013 meher (%, yes)
Full package	0.664***	0.750***	0.291***	0.037	0.325***
	(0.046)	(0.051)	(0.043)	(0.047)	(0.057)
Marketing assistance	−0.051	−0.003	−0.002	−0.004	0.042
	(0.047)	(0.045)	(0.034)	(0.040)	(0.057)
Model farmer	0.054	0.055	0.014	0.064	0.040
	(0.055)	(0.054)	(0.041)	(0.049)	(0.066)
Female farmer	0.132	0.201**	−0.059	−0.062	0.039
	(0.097)	(0.095)	(0.056)	(0.073)	(0.104)
Treatment × model	−0.062	−0.111	0.013	−0.039	0.055
	(0.074)	(0.080)	(0.065)	(0.074)	(0.083)
Treatment × female	−0.120	−0.276**	0.053	0.070	0.044
	(0.107)	(0.118)	(0.084)	(0.097)	(0.117)
Other control Variables?	Yes	Yes	Yes	Yes	Yes
Kebele fixed effect	Yes	Yes	Yes	Yes	Yes
Control group mean	29.3	16.7	4.7	13.1	49.1
Observations	490	490	490	490	490
*R* ^2^	0.537	0.500	0.408	0.203	0.306

*Source*: Authors’ calculation based on data from the 2014 wheat growers’ survey. Robust standard errors in parentheses. ***P < 0.01, **P < 0.05, *P < 0.1. Full results available in Supporting Information Appendix Table A.4.

At this point, almost all the coefficient estimates on the marketing group are not different from zero, and column 4 suggests good explanatory power. Yet neither the full‐package nor the marketing assistance group farmers were more likely to receive marketing assistance than the control group. As only 13% of the control group reported receiving marketing assistance, this component of the intervention clearly did not occur as planned.

Lastly, consistent with expectations, a significant percentage of full‐package farmers report growing wheat differently during the 2013 *meher* season (column 5). The marketing‐group farmers were no different from the control farmers in terms of their access to the components of the Wheat Initiative package, which is plausible except for the marketing assistant component. Overall, implementation of the wheat package seems to have functioned through higher access to inputs and some change in techniques, while marketing assistance services were poorly implemented.

#### 5.3.3. Adoption of agricultural technologies

Along with inputs, the Wheat Initiative suggested the adoption of specific practices. We next explore whether farmers used specific inputs, the seeding and fertilizer rates, and whether farmers used row planting (Table 8). Almost all full‐package farmers planted certified wheat seeds, and nearly all applied both urea and DAP fertilizers on their experimental plots. We find a 50 percentage point difference in certified seed use over the control group, and an 11.8 percentage point difference for urea. Likewise, the full‐package farmers planted about 24 kg fewer seeds per hectare than the farmers in the marketing assistance and control groups (column 2). Although fertilizer use rates were higher in all three groups than the Ethiopia‐wide averages reported in Section 2, they remain slightly lower than the recommended 200 kg/ha. Further, full‐package farmers were more likely to use row planting (column 7) than marketing assistance and control groups, albeit not many of them did. Qualitative observations suggested that some full‐package farmers did not use row planting because of the extra labor requirement, while others felt that row planting was impractical because they had “black cotton” soils. Overall, as indicated above, the main effect of the Wheat Initiative seems related to enhanced access to seeds and fertilizers. There appears to also be some movement toward a lower seeding rate and row planting, though not all full‐package farmers followed these recommendations.

**Table 8:** Farmers’ implementation of the promotional wheat package

Variables	Certified seed (%, yes)	Certified seed quantity (kg/ha)	Urea (%, yes)	Urea applied (kg/ha)	DAP (%, yes)	DAP applied (kg/ha)	Row planting (%, yes)
Full package	0.490***	−22.783*	0.113***	15.774	−0.002	−3.910	0.367***
	(0.048)	(11.726)	(0.028)	(11.905)	(0.003)	(9.867)	(0.051)
Marketing assistance	0.009	−7.885	0.003	−4.306	−0.009	−11.249	0.003
	(0.054)	(10.504)	(0.028)	(8.770)	(0.009)	(8.737)	(0.047)
Model farmer	0.105*	−15.719	0.031	−1.432	−0.015	6.824	0.060
	(0.064)	(11.726)	(0.031)	(9.407)	(0.014)	(10.269)	(0.056)
Female farmer	0.109	−36.069*	0.034	−2.844	−0.001	−5.872	0.005
	(0.095)	(19.039)	(0.037)	(15.804)	(0.004)	(12.227)	(0.075)
Treatment × model	−0.122*	−2.094	−0.056	5.820	−0.002	−7.685	−0.102
	(0.073)	(14.898)	(0.040)	(17.033)	(0.016)	(16.303)	(0.075)
Treatment × female	−0.088	27.171	−0.039	18.847	0.004	11.746	−0.037
	(0.101)	(19.285)	(0.043)	(21.907)	(0.007)	(17.414)	(0.093)
Other control variables?	Yes	Yes	Yes	Yes	Yes	Yes	Yes
Kebele fixed effect	Yes	Yes	Yes	Yes	Yes	Yes	Yes
Control group mean	51.5	177.9	91.0	129.6	100.0	153.0	26.9
Observations	490	346	490	464	490	488	490
*R* ^2^	0.398	0.358	0.288	0.479	0.097	0.502	0.502

*Source*: Authors’ calculations based on the 2014 wheat growers’ survey. Robust standard errors in parentheses. ***P < 0.01, **P < 0.05, *P < 0.1. Full results in Supporting Information Appendix Table A.5.

#### 5.3.4. Sustained adoption

The success of the Wheat Initiative package ultimately depends on full‐package adoption both benchmark farmers and neighboring farmers even after subsidies are removed. Table 9 presents farmer plans for the following wheat‐growing season, focusing on practices recommended by the Wheat Initiative. Although 80% of farmers stated their willingness to plant certified wheat seeds for the next season, even if they have to pay cash for it, there is no statistically significant difference between plans among full‐package and control group farmers (columns 1 and 2). However, full‐package farmers are 18.6 percentage points more likely to adopt row planting compared to farmers in marketing‐only and control groups (column 3). Farmers indicated that hand broadcasting reduces labor requirements for seeding. Similar to row planting, full‐package farmers are more likely to state they will reduce the seeding rate relative to control group farmers (column 4). Farmers deciding to maintain the traditional seeding rate indicated that they are unsure whether the reduced seeding rate increases yields. About half of the farmers plan to use recommended fertilizer application rates in the coming season, with no significant differences between groups. In sum, these results suggest Wheat Initiative techniques may be gradually adopted.

**Table 9:** Farmers’ plans for adopting the promotional wheat package in the 2014 *meher* season

Variables	Plan to buy/apply …				
	Seed if on cash (%, yes)	Seed if on credit (%, yes)	Row planting	Reduced seeding rate	Recommended (more) fertilizer
Full package	−0.016	−0.075	0.184***	0.074**	0.105
	(0.047)	(0.056)	(0.055)	(0.030)	(0.064)
Marketing assistance	−0.038	−0.064	0.081	0.008	0.023
	(0.043)	(0.047)	(0.050)	(0.034)	(0.056)
Model farmer	−0.005	−0.110*	0.004	0.028	−0.051
	(0.050)	(0.060)	(0.060)	(0.040)	(0.069)
Female farmer	−0.065	−0.024	0.069	−0.004	−0.069
	(0.090)	(0.082)	(0.084)	(0.068)	(0.087)
Treatment × model	−0.060	−0.038	−0.110	−0.072	−0.038
	(0.076)	(0.092)	(0.086)	(0.053)	(0.094)
Treatment × female	0.057	0.074	−0.142	0.007	−0.067
	(0.114)	(0.107)	(0.112)	(0.076)	(0.119)
Other control variables?	Yes	Yes	Yes	Yes	Yes
Kebele fixed effect	Yes	Yes	Yes	Yes	Yes
Control group mean	85.6	82.6	28.7	89.8	45.5
Observations	490	490	490	490	490
*R* ^2^	0.146	0.238	0.346	0.136	0.255

*Source*: Authors’ calculations based on the 2014 wheat growers’ survey. Robust standard errors in parentheses. ***P < 0.01, **P < 0.05, *P < 0.1. Full results in Supporting Information Appendix Table A.6.

## 6. Conclusion

Package interventions are potentially attractive ways to try to overcome multiple adoption constraints at once. In this article, we evaluate the impacts of the Ethiopian ATA Wheat Initiative on wheat yields, using three different measures of yields. Starting with a list of 14 farmers each in 36 *kebeles*, farmers were randomly allocated the farmers into two treatment groups, a full‐package group, a marketing‐only group, and a control group. We find that the full‐package intervention had a 14% increase on wheat yields, measured with both crop‐cuts and farmer predicted yields, once we control for farmer type and household characteristics. Farmer recall data showed a smaller and statistically insignificant yield increase, which is probably attributable to measurement error in plot size, demonstrating that one should use data on farmer reports of plot size and production cautiously when computing yields.

We then trace how farmers actually implemented components of the Wheat Initiative. Farmers receiving the full package were much more likely to use certified seeds and received quality fertilizer from the Wheat Initiative than the control group; however, in both cases we find leakage of subsidized inputs to the control group, implying that impact estimates for the package as implemented may be somewhat underestimated. Full‐package farmers were far more likely to apply gypsum to their fields than assistance or control group farmers, but no more likely to use pesticides or herbicides.

In terms of techniques, we find that full‐package farmers reduced seeding rates and were more likely to try row planting than control group farmers. Although most farmers in all groups were aware of new recommendations, not all full‐package farmers followed recommendations about techniques, and while they reduced the seeding rate, they did not reduce it as much as recommended. Full‐package farmers were more likely to suggest they would reduce seeding rates and increase row planting in the following season, but differences between the full‐package group and the control group were small. So, changing material input rates when they are made available is not that difficult, whereas changing farmer behavior takes more time, and likely contributes to the difference between findings from agronomic trials that suggest substantial yield increases, and results from real‐life randomised control trials (RCTs) like this one. That said, to the extent that farmers learn how to use these practices over time, yield increases may continue in the future. Yet many farmers may not adopt row planting, due to the implicit cost of additional labor to row plant. When planning package interventions, it is important to consider how labor allocations would need to change as farmers may be averse to adopting labor‐intensive practices.

Finally, the marketing assistance was effectively not implemented. Only 13–16% of farmers reported receiving marketing assistance. Ensuring that farmers can link to markets to sell excess production would further reduce uncertainty among farmers about their potential profits and could also stimulate the use of improved agronomic practices; this hypothesis deserves further attention in future research.

Important lessons for both intervention design and policy can be gleaned from this study. From a design perspective, the drawback of using a “package” is that we do not know which package components contributed most to the yield increase, and which components could have been either minimized or dropped altogether. Additive designs can help shed light on how important different pieces of such packages are to attaining goals; however, sample size requirements for such studies can be substantial when testing more than two elements of a package, as even with three elements one needs six treatment groups and a control group. From a policy perspective, the results show that intensification through the promotion of such packages is quite possible, but expectations about increases in productivity that would be observed will necessarily lag substantially behind those of agronomic trials in experimental stations.

## Supporting information


**Appendix Table A.1**: Average Yields by Crop‐Cut Status
**Appendix Table A.2**.: Power Calculations for Realized Differences in Averages
**Appendix Table A.3**: Farmers’ knowledge of the promotional wheat package
**Appendix Table A.4**: Farmers’ experiences with the services provided under the Wheat Initiative
**Appendix Table A.5**: Farmers’ implementation of the promotional wheat package
**Appendix Table A.6**: Farmers’ plans for adopting the promotional wheat package in the following season (2014 *meher* season)
**Appendix Table A.7**: Farmers knowledge of the promotional wheat package
**Appendix Table A.8**: Farmers experiences with the services provided under the Wheat Initiative
**Appendix Table A.9**: Farmers implementation of the promotional wheat package
**Appendix Table A.10**: Farmers plans for adopting the promotional wheat package in the following season (2014 meher season)
**Appendix Table A.11**: Characterizing nonresponses in crop‐cut production

Supporting Information

Supporting Information

Supporting Information

## Footnotes

1 A *kebele* is the smallest administrative unit in Ethiopia.

2 A similar study by Vandercasteelen et al. (2013) studies the ATA's introduction of a similar package to increase teff yields; they find a 2% yield gain from row planting based on crop‐cut data, and a 12–13% yield gain based on the data from farmers’ production and area estimates, also using a randomized control trial.

3 Certified seed refers to improved seed newly purchased or received by farmers, which was not retained from their own harvest or exchanged with neighboring farmers. Certified seed is produced to ensure physical quality and genetic integrity, and from the farmer perspective it may either be a new variety or just fresh seed of a previously grown variety.

4 The System of Wheat Intensification is a method of cultivating wheat integrating agronomic principles with a package of practices in wheat crop production. The promoted package includes the use of improved and treated seed, land preparation with increased use of organic matters, lower seeding rate, row planting, gap filling, (irrigation), and using a mechanical weeder for better soil aeration (Dhar et al., 2016). The System of Wheat Intensification is closely related to the somewhat controversial System of Rice Intensification (Glover, 2011).

5 A model farmer is a lead farmer that is a risk taker and receptive to new ideas, technologies, and practices and works closely with extension agents to spread and impart information and promoting technologies to neighboring farmers.

6 EGTE's plan to purchase wheat from smallholders was publicized widely around planting time, but the guarantee was specific to benchmark farmers and to market assistance only farmers, described next.

7 Overall, each *woreda* included between 4 and 10 *kebeles*.

8 Outputs could not be measured for the remaining 122 plots for three reasons: seven farmers had no wheat plot during the 2013 *meher* season, five farmers could not be identified by anyone in the *kebele* during the survey, and the remaining 110 farmers harvested their wheat plots prior to the crop‐cut.

9 Supporting Information Appendix Table A.2 discusses the implications of our realized sample size for statistical power. We describe realized values of the mean logarithm of yield outcomes, the standard deviations of those outcomes, and the sample size for each of the three available measures. We then compute the statistical power for the realized sample, meaning the probability that the measured difference would be found significant at the 5% level, under the assumption that differences in average yields are population parameters.
