# Supplementary material for: The impact of the use of new technologies on farmers’ wheat yield in Ethiopia: evidence from a randomized control trial
Source: Agric Econ. 2018 Jul 2;49(4):409–21. doi: 10.1111/agec.12425 (PMC6108534; doi:10.1111/agec.12425)
Supplement: Supplementary file 1 — Appendix Table A.1: Average Yields by Crop‐Cut Status Appendix Table A.2.: Power Calculations for Realized Differences in Averages Appendix Table A.3: Farmers’ knowledge of the promotional wheat package Appendix Table A.4: Farmers’ experiences with the services provided under the Wheat Initiative Appendix Table A.5: Farmers’ implementation of the promotional wheat package Appendix Table A.6: Farmers’ plans for adopting the promotional wheat package in the following season (2014 meher season) Appendix Table A.7: Farmers knowledge of the promotional wheat package Appendix Table A.8: Farmers experiences with the services provided under the Wheat Initiative Appendix Table A.9: Farmers implementation of the promotional wheat package Appendix Table A.10: Farmers plans for adopting the promotional wheat package in the following season (2014 meher season) Appendix Table A.11: Characterizing nonresponses in crop‐cut production [file AGEC-49-409-s001.docx]

**Appendix**

Appendix Table A.1. Average Yields by Crop-Cut Status

|  | Sample farmers with crop-cut data (n=367) | | Sample farmers without crop-cut data (n=122) | |
| --- | --- | --- | --- | --- |
|  | Mean | Std. Error | Mean | Std. Error |
| Predicted yield | 3.11*** | 0.08 | 4.37 | 0.16 |
| Recall yield | 2.73*** | 0.08 | 4.06 | 0.18 |

Appendix Table A.2.: Power Calculations for Realized Differences in Averages

| Outcome | Average, Treatment | Average, Control | Std. Deviation, Treatment | Std. Deviation, Control | Number of Obs., treatment | Number of Obs. control | Power to detect difference at 5 percent level |
| --- | --- | --- | --- | --- | --- | --- | --- |
| *Panel A (full package vs control)* | | | | | | | |
| Yield: crop-cut | 7.802 | 7.735 | 0.6645 | 0.6546 | 124 | 148 | 0.132 |
| Yield: predicted | 8.069 | 7.917 | 0.5780 | 0.6308 | 194 | 163 | 0.653 |
| Yield: recall | 7.869 | 7.779 | 0.6940 | 0.6817 | 197 | 166 | 0.238 |
| *Panel B (market vs control)* | | | | | | | |
| Yield: crop-cut | 7.785 | 7.735 | 0.5854 | 0.6546 | 95 | 148 | 0.095 |
| Yield: predicted | 7.943 | 7.917 | 0.5806 | 0.6308 | 125 | 163 | 0.066 |
| Yield: recall | 7.828 | 7.779 | 0.7221 | 0.6817 | 126 | 166 | 0.091 |

**Appendix Table A.3. Farmers’ knowledge of the promotional wheat package**

|  | Information on ATA Wheat initiative *(%, yes)* | Training on wheat production method *(%, yes)* | Package include certified seed *(%, yes)* | Package include reduced seed rate *(%, yes)* | Package include row planting *(%, yes)* | Urea application rate *(kg/ha)* | DAP application rate *(kg/ha)* |
| --- | --- | --- | --- | --- | --- | --- | --- |
| Variables |  |  |  |  |  |  |  |
| Full package | 0.382*** | 0.589*** | 0.011 | 0.062 | 0.002 | -1.679 | -4.682 |
|  | (0.051) | (0.052) | (0.018) | (0.045) | (0.043) | (8.148) | (8.987) |
| Marketing assistance | -0.007 | 0.106* | 0.023 | -0.027 | -0.074* | 3.600 | -1.863 |
|  | (0.061) | (0.056) | (0.016) | (0.047) | (0.039) | (6.953) | (7.140) |
| Model farmer | 0.061 | 0.228*** | 0.017* | 0.091* | 0.053 | -5.776 | -4.631 |
|  | (0.069) | (0.070) | (0.010) | (0.048) | (0.037) | (9.835) | (8.128) |
| Female farmer | -0.004 | 0.034 | -0.078 | -0.066 | -0.052 | 2.014 | 1.857 |
|  | (0.100) | (0.085) | (0.058) | (0.098) | (0.084) | (8.280) | (9.231) |
| Treatment × model | -0.094 | -0.165** | -0.004 | -0.047 | -0.031 | 19.409 | 4.839 |
|  | (0.083) | (0.083) | (0.015) | (0.067) | (0.060) | (12.095) | (11.556) |
| Treatment × female | 0.065 | -0.059 | 0.089 | -0.003 | 0.050 | 0.865 | 5.479 |
|  | (0.108) | (0.102) | (0.058) | (0.117) | (0.099) | (11.332) | (13.103) |
| Age of household head | -0.002 | 0.003 | -0.000 | 0.001 | 0.000 | 0.048 | -0.101 |
|  | (0.002) | (0.002) | (0.001) | (0.002) | (0.002) | (0.298) | (0.318) |
| Education of household head | -0.006 | 0.017 | -0.011 | 0.017 | 0.014 | 3.639 | 2.996 |
|  | (0.026) | (0.025) | (0.010) | (0.025) | (0.018) | (3.526) | (4.138) |
| Landholding size | -0.004 | -0.009 | -0.001 | -0.014 | 0.009 | -1.254 | -1.933 |
|  | (0.015) | (0.012) | (0.002) | (0.010) | (0.009) | (1.397) | (1.407) |
| Household size | 0.010 | 0.006 | -0.000 | -0.004 | 0.000 | 0.562 | 1.244 |
|  | (0.010) | (0.009) | (0.003) | (0.008) | (0.007) | (1.053) | (1.157) |
| Black soil | 0.031 | -0.115** | 0.003 | -0.063 | 0.002 | -2.663 | -8.778 |
|  | (0.057) | (0.055) | (0.018) | (0.054) | (0.043) | (7.543) | (6.939) |
| Gray/sandy soil | -0.005 | -0.170** | 0.016 | -0.024 | 0.005 | -4.135 | -1.837 |
|  | (0.067) | (0.066) | (0.016) | (0.054) | (0.048) | (8.536) | (9.056) |
| Distance to plot | -0.001 | 0.000 | 0.000 | 0.001 | -0.002 | 0.335 | 0.181 |
|  | (0.002) | (0.001) | (0.000) | (0.002) | (0.001) | (0.233) | (0.262) |
| Radio ownership | 0.109** | 0.015 | 0.014 | -0.032 | 0.028 | 3.415 | 1.093 |
|  | (0.051) | (0.047) | (0.018) | (0.043) | (0.040) | (6.486) | (7.435) |
| Television ownership | -0.082 | -0.050 | -0.020 | -0.016 | -0.027 | -12.186* | -20.421** |
|  | (0.066) | (0.060) | (0.023) | (0.047) | (0.044) | (7.336) | (8.933) |
| Cellphone ownership | 0.021 | 0.017 | -0.000 | -0.058 | -0.037 | -10.144 | -5.838 |
|  | (0.061) | (0.057) | (0.023) | (0.049) | (0.045) | (8.994) | (8.357) |
| Bicycle ownership | 0.018 | 0.194* | 0.001 | -0.030 | -0.089 | 8.751 | 18.194 |
|  | (0.113) | (0.106) | (0.013) | (0.063) | (0.086) | (20.331) | (18.649) |
| Car ownership | -0.059 | -0.280** | -0.004 | -0.037 | -0.020 | 15.545 | 3.228 |
|  | (0.111) | (0.114) | (0.012) | (0.060) | (0.062) | (9.639) | (14.651) |
| Livestock ownership (in TLU) | 0.001 | -0.001 | 0.000 | 0.002 | 0.000 | 0.015 | 0.615 |
|  | (0.003) | (0.003) | (0.001) | (0.002) | (0.002) | (0.357) | (0.473) |
| Constant | 0.524*** | 0.201 | 0.993*** | 0.891*** | 0.829*** | 111.534*** | 138.363*** |
|  | (0.168) | (0.148) | (0.052) | (0.142) | (0.113) | (23.322) | (27.033) |
| Kebele fixed effect | Yes | Yes | Yes | Yes | Yes | Yes | Yes |
| Mean for control group | 57.4 | 38.3 | 94.4 | 87.9 | 91.1 | 163.5 | 185.3 |
| Observations | 469 | 469 | 451 | 469 | 469 | 428 | 430 |
| R-squared | 0.260 | 0.425 | 0.112 | 0.185 | 0.416 | 0.544 | 0.584 |

Source: Authors’ calculation based on data from the 2014 wheat growers’ survey. Robust standard errors in parentheses. *** p<0.01, ** p<0.05, * p<0.1.

**Appendix Table A.4. Farmers’ experiences with the services provided under the Wheat Initiative**

|  | Received certified seed *(%, yes on time)* | Quality of seed *(%, very good)* | Received Urea for free *(%, yes on time)* | Quality of Urea *(%, very good)* | Received gypsum for free *(%, yes on time)* | Received marketing assistance *(%, yes)* | Grow wheat differently in 2013 meher *(%, yes)* | Know a friend/neighbor grow wheat differently in 2013 meher *(%, yes)* |
| --- | --- | --- | --- | --- | --- | --- | --- | --- |
| Variables |  |  |  |  |  |  |  |  |
| Full package | 0.664*** | 0.460*** | 0.750*** | 0.480*** | 0.291*** | 0.037 | 0.325*** | 0.040 |
|  | (0.046) | (0.061) | (0.051) | (0.056) | (0.043) | (0.047) | (0.057) | (0.056) |
| Marketing assistance | -0.051 | 0.025 | -0.003 | 0.017 | -0.002 | -0.004 | 0.042 | -0.089* |
|  | (0.047) | (0.048) | (0.045) | (0.037) | (0.034) | (0.040) | (0.057) | (0.050) |
| Model farmer | 0.054 | 0.066 | 0.055 | 0.043 | 0.014 | 0.064 | 0.040 | -0.041 |
|  | (0.055) | (0.059) | (0.054) | (0.044) | (0.041) | (0.049) | (0.066) | (0.058) |
| Female farmer | 0.132 | -0.004 | 0.201** | 0.020 | -0.059 | -0.062 | 0.039 | 0.115 |
|  | (0.097) | (0.086) | (0.095) | (0.061) | (0.056) | (0.073) | (0.104) | (0.079) |
| Treatment × model | -0.062 | -0.041 | -0.111 | 0.014 | 0.013 | -0.039 | 0.055 | 0.055 |
|  | (0.074) | (0.093) | (0.080) | (0.084) | (0.065) | (0.074) | (0.083) | (0.082) |
| Treatment × female | -0.120 | 0.118 | -0.276** | -0.032 | 0.053 | 0.070 | 0.044 | -0.106 |
|  | (0.107) | (0.121) | (0.118) | (0.108) | (0.084) | (0.097) | (0.117) | (0.103) |
| Age of household head | 0.001 | 0.004 | 0.002 | 0.004* | -0.000 | -0.000 | -0.002 | 0.002 |
|  | (0.002) | (0.002) | (0.002) | (0.002) | (0.002) | (0.002) | (0.002) | (0.002) |
| Education of household head | 0.020 | 0.006 | 0.011 | 0.006 | 0.002 | -0.033* | 0.003 | 0.008 |
|  | (0.022) | (0.024) | (0.024) | (0.020) | (0.018) | (0.019) | (0.026) | (0.026) |
| Landholding size | -0.009 | -0.019 | -0.006 | -0.019 | 0.023** | 0.009 | 0.011 | 0.018 |
|  | (0.012) | (0.014) | (0.013) | (0.012) | (0.010) | (0.010) | (0.013) | (0.013) |
| Household size | -0.006 | -0.006 | -0.003 | 0.002 | 0.002 | 0.005 | -0.003 | -0.002 |
|  | (0.009) | (0.011) | (0.009) | (0.009) | (0.007) | (0.008) | (0.010) | (0.009) |
| Black soil | 0.015 | 0.029 | 0.027 | -0.012 | 0.032 | -0.023 | -0.021 | -0.029 |
|  | (0.051) | (0.059) | (0.053) | (0.051) | (0.044) | (0.047) | (0.058) | (0.060) |
| Gray/sandy soil | -0.061 | -0.032 | 0.028 | -0.014 | 0.062 | 0.054 | -0.061 | -0.042 |
|  | (0.055) | (0.064) | (0.059) | (0.056) | (0.050) | (0.050) | (0.066) | (0.065) |
| Distance to plot | 0.000 | -0.002 | -0.001 | 0.000 | -0.001 | -0.000 | 0.001 | -0.000 |
|  | (0.001) | (0.001) | (0.001) | (0.001) | (0.001) | (0.001) | (0.001) | (0.002) |
| Radio ownership | 0.005 | 0.036 | 0.017 | 0.033 | -0.001 | 0.063 | -0.097* | 0.106** |
|  | (0.043) | (0.050) | (0.041) | (0.042) | (0.036) | (0.039) | (0.050) | (0.049) |
| Television ownership | 0.029 | 0.004 | -0.041 | 0.070 | -0.085* | 0.069 | 0.009 | -0.011 |
|  | (0.056) | (0.065) | (0.059) | (0.058) | (0.043) | (0.062) | (0.069) | (0.060) |
| Cellphone ownership | -0.040 | -0.044 | -0.115** | -0.074 | 0.024 | 0.025 | 0.105* | 0.015 |
|  | (0.049) | (0.055) | (0.050) | (0.046) | (0.042) | (0.045) | (0.058) | (0.055) |
| Bicycle ownership | -0.030 | -0.025 | 0.080 | -0.069 | 0.096 | -0.088 | -0.265*** | -0.185 |
|  | (0.114) | (0.114) | (0.104) | (0.093) | (0.076) | (0.074) | (0.099) | (0.122) |
| Car ownership | -0.111 | -0.097 | 0.023 | -0.024 | -0.056 | -0.145 | -0.104 | 0.035 |
|  | (0.079) | (0.094) | (0.092) | (0.090) | (0.067) | (0.111) | (0.099) | (0.077) |
| Livestock ownership (in TLU) | -0.000 | 0.002 | 0.000 | -0.002 | -0.003 | -0.004 | 0.004 | -0.001 |
|  | (0.003) | (0.004) | (0.003) | (0.004) | (0.003) | (0.003) | (0.003) | (0.004) |
| Kebele fixed effect | Yes | Yes | Yes | Yes | Yes | Yes | Yes | Yes |
| Mean for control group | 29.3 | 20.9 | 16.7 | 32.1 | 4.7 | 13.1 | 49.1 | 76.6 |
| Observations | 490 | 490 | 490 | 490 | 490 | 490 | 490 | 490 |
| R-squared | 0.537 | 0.336 | 0.500 | 0.369 | 0.408 | 0.203 | 0.306 | 0.226 |

Source: Authors’ calculation based on data from the 2014 wheat growers’ survey. Robust standard errors in parentheses. *** p<0.01, ** p<0.05, * p<0.1

**Appendix Table A.5. Farmers’ implementation of the promotional wheat package**

|  | Certified seed *(%, yes)* | Certified seed quantity *(kg/ha)* | Urea *(%, yes)* | Urea applied *(kg/ha)* | DAP *(%, yes)* | DAP applied *(kg/ha)* | Gypsum *(%, yes)* | Pesticide *(%, yes)* | Herbicide *(%, yes)* | Row planting *(%, yes)* |
| --- | --- | --- | --- | --- | --- | --- | --- | --- | --- | --- |
| Variables |  |  |  |  |  |  |  |  |  |  |
| Full package | 0.490*** | -22.783* | 0.113*** | 15.774 | -0.002 | -3.910 | 0.281*** | 0.006 | -0.036 | 0.367*** |
|  | (0.048) | (11.726) | (0.028) | (11.905) | (0.003) | (9.867) | (0.043) | (0.041) | (0.036) | (0.051) |
| Marketing assistance | 0.009 | -7.885 | 0.003 | -4.306 | -0.009 | -11.249 | 0.009 | 0.027 | -0.010 | 0.003 |
|  | (0.054) | (10.504) | (0.028) | (8.770) | (0.009) | (8.737) | (0.034) | (0.036) | (0.038) | (0.047) |
| Model farmer | 0.105* | -15.719 | 0.031 | -1.432 | -0.015 | 6.824 | 0.013 | -0.001 | 0.022 | 0.060 |
|  | (0.064) | (11.726) | (0.031) | (9.407) | (0.014) | (10.269) | (0.042) | (0.043) | (0.050) | (0.056) |
| Female farmer | 0.109 | -36.069* | 0.034 | -2.844 | -0.001 | -5.872 | -0.062 | -0.038 | 0.116* | 0.005 |
|  | (0.095) | (19.039) | (0.037) | (15.804) | (0.004) | (12.227) | (0.057) | (0.032) | (0.067) | (0.075) |
| Treatment × model | -0.122* | -2.094 | -0.056 | 5.820 | -0.002 | -7.685 | 0.024 | 0.024 | -0.022 | -0.102 |
|  | (0.073) | (14.898) | (0.040) | (17.033) | (0.016) | (16.303) | (0.065) | (0.062) | (0.066) | (0.075) |
| Treatment × female | -0.088 | 27.171 | -0.039 | 18.847 | 0.004 | 11.746 | 0.047 | 0.095 | -0.073 | -0.037 |
|  | (0.101) | (19.285) | (0.043) | (21.907) | (0.007) | (17.414) | (0.082) | (0.066) | (0.080) | (0.093) |
| Age of household head | 0.001 | -0.250 | 0.000 | -0.165 | -0.000 | -0.173 | 0.000 | -0.001 | -0.002 | -0.003 |
|  | (0.002) | (0.409) | (0.001) | (0.367) | (0.000) | (0.373) | (0.002) | (0.002) | (0.001) | (0.002) |
| Education of household head | 0.013 | -8.699* | 0.011 | -3.392 | 0.000 | 2.230 | 0.000 | -0.000 | -0.007 | 0.001 |
|  | (0.023) | (5.100) | (0.013) | (4.558) | (0.004) | (4.176) | (0.017) | (0.018) | (0.017) | (0.023) |
| Landholding size | 0.003 | -6.946*** | 0.007 | -4.110** | 0.003 | -5.701*** | 0.018* | 0.012 | -0.001 | 0.012 |
|  | (0.014) | (2.329) | (0.007) | (1.887) | (0.003) | (2.161) | (0.010) | (0.014) | (0.008) | (0.013) |
| Household size | -0.017* | -0.932 | -0.007 | -3.450** | -0.000 | -2.168 | 0.000 | -0.002 | 0.005 | 0.005 |
|  | (0.009) | (1.767) | (0.006) | (1.730) | (0.001) | (1.532) | (0.007) | (0.008) | (0.006) | (0.009) |
| Black soil | -0.092* | 0.485 | 0.012 | 13.419 | 0.005 | 8.665 | -0.006 | -0.031 | 0.021 | -0.078 |
|  | (0.049) | (10.126) | (0.026) | (11.174) | (0.006) | (10.619) | (0.045) | (0.037) | (0.042) | (0.049) |
| Gray/sandy soil | -0.106* | 17.485 | 0.013 | 2.245 | 0.011 | 15.259 | 0.025 | -0.055 | 0.035 | 0.011 |
|  | (0.057) | (13.245) | (0.032) | (13.004) | (0.008) | (13.615) | (0.049) | (0.042) | (0.049) | (0.059) |
| Distance to plot | 0.000 | -0.252 | -0.000 | -0.632** | 0.000 | -0.560** | -0.001 | 0.000 | -0.000 | 0.001 |
|  | (0.001) | (0.360) | (0.001) | (0.302) | (0.000) | (0.272) | (0.001) | (0.001) | (0.001) | (0.001) |
| Radio ownership | -0.057 | 8.159 | -0.027 | -11.285 | -0.008 | 0.444 | 0.005 | 0.007 | -0.010 | 0.096** |
|  | (0.042) | (9.236) | (0.022) | (9.563) | (0.008) | (8.455) | (0.036) | (0.036) | (0.035) | (0.042) |
| Television ownership | -0.011 | 23.813** | -0.072* | -9.134 | 0.008 | 2.894 | -0.069 | 0.007 | -0.014 | -0.013 |
|  | (0.063) | (10.614) | (0.042) | (10.466) | (0.009) | (13.827) | (0.045) | (0.053) | (0.046) | (0.060) |
| Cellphone ownership | 0.063 | -20.877* | -0.005 | 3.403 | 0.007 | 2.656 | 0.017 | 0.043 | 0.052 | 0.001 |
|  | (0.050) | (12.098) | (0.025) | (12.055) | (0.008) | (10.003) | (0.042) | (0.031) | (0.037) | (0.047) |
| Bicycle ownership | 0.100 | -12.238 | -0.003 | 21.689 | 0.012 | 14.967 | 0.096 | -0.048 | 0.079 | -0.051 |
|  | (0.119) | (14.920) | (0.098) | (17.605) | (0.011) | (17.260) | (0.075) | (0.090) | (0.077) | (0.105) |
| Car ownership | -0.141 | -9.889 | -0.171 | -0.871 | 0.001 | 0.752 | -0.061 | 0.096 | 0.025 | -0.044 |
|  | (0.108) | (17.079) | (0.106) | (12.691) | (0.009) | (15.715) | (0.066) | (0.134) | (0.043) | (0.116) |
| Livestock ownership (in TLU) | 0.006** | 1.740*** | 0.003 | 1.237** | -0.000 | 1.623*** | -0.002 | 0.000 | -0.000 | -0.002 |
|  | (0.003) | (0.601) | (0.002) | (0.535) | (0.000) | (0.592) | (0.003) | (0.003) | (0.002) | (0.004) |
| Kebele fixed effect | Yes | Yes | Yes | Yes | Yes | Yes | Yes | Yes | Yes | Yes |
| Mean for control group | 51.5 | 177.9 | 91.0 | 129.6 | 100.0 | 153.0 | 4.7 | 8.9 | 59.8 | 26.9 |
| Observations | 490 | 346 | 490 | 464 | 490 | 488 | 490 | 490 | 490 | 490 |
| R-squared | 0.398 | 0.358 | 0.288 | 0.479 | 0.097 | 0.502 | 0.407 | 0.262 | 0.680 | 0.502 |

Source: Authors’ calculation based on data from the 2014 wheat growers’ survey. Robust standard errors in parentheses. *** p<0.01, ** p<0.05, * p<0.1

**Appendix Table A.6. Farmers’ plans for adopting the promotional wheat package in the following season (2014 *meher* season)**

|  | Plan to buy/apply . . . | | | | |
| --- | --- | --- | --- | --- | --- |
| Variables | Seed if on cash *(%, yes)* | Seed if on credit *(%, yes)* | Row planting | Reduced seeding rate | Recommended (more) fertilizer |
| Full package | -0.016 | -0.075 | 0.184*** | 0.074** | 0.102 |
|  | (0.047) | (0.056) | (0.055) | (0.030) | (0.064) |
| Marketing assistance | -0.038 | -0.064 | 0.081 | 0.008 | 0.023 |
|  | (0.043) | (0.047) | (0.050) | (0.034) | (0.056) |
| Model farmer | -0.005 | -0.110* | 0.004 | 0.028 | -0.050 |
|  | (0.050) | (0.060) | (0.060) | (0.040) | (0.069) |
| Female farmer | -0.065 | -0.024 | 0.069 | -0.004 | -0.066 |
|  | (0.090) | (0.082) | (0.084) | (0.068) | (0.086) |
| Treatment × model | -0.060 | -0.038 | -0.110 | -0.072 | -0.054 |
|  | (0.076) | (0.092) | (0.086) | (0.053) | (0.094) |
| Treatment × female | 0.057 | 0.074 | -0.142 | 0.007 | -0.063 |
|  | (0.114) | (0.107) | (0.112) | (0.076) | (0.118) |
| Age of household head | -0.001 | -0.004* | -0.003 | -0.004*** | -0.006** |
|  | (0.002) | (0.002) | (0.002) | (0.002) | (0.002) |
| Education of household head | 0.044* | 0.016 | 0.013 | 0.011 | -0.040 |
|  | (0.023) | (0.023) | (0.023) | (0.017) | (0.027) |
| Landholding size | -0.004 | -0.004 | 0.004 | 0.007 | 0.013 |
|  | (0.010) | (0.013) | (0.014) | (0.008) | (0.017) |
| Household size | -0.001 | 0.025*** | 0.013 | 0.017*** | 0.028** |
|  | (0.008) | (0.010) | (0.010) | (0.007) | (0.011) |
| Black soil | 0.004 | -0.130** | 0.042 | -0.026 | 0.068 |
|  | (0.053) | (0.051) | (0.053) | (0.031) | (0.058) |
| Gray/sandy soil | 0.068 | -0.144** | 0.074 | -0.056 | -0.036 |
|  | (0.055) | (0.060) | (0.063) | (0.038) | (0.068) |
| Distance to plot | -0.000 | 0.001 | -0.001 | -0.000 | 0.002 |
|  | (0.001) | (0.002) | (0.001) | (0.001) | (0.002) |
| Radio ownership | 0.015 | -0.017 | -0.017 | 0.008 | 0.010 |
|  | (0.043) | (0.044) | (0.050) | (0.033) | (0.055) |
| Television ownership | 0.047 | 0.031 | -0.036 | -0.056 | 0.034 |
|  | (0.055) | (0.066) | (0.075) | (0.052) | (0.079) |
| Cellphone ownership | -0.023 | 0.002 | 0.024 | 0.073* | -0.053 |
|  | (0.050) | (0.053) | (0.056) | (0.039) | (0.059) |
| Bicycle ownership | -0.063 | 0.017 | 0.023 | 0.047 | 0.258** |
|  | (0.083) | (0.111) | (0.126) | (0.063) | (0.118) |
| Car ownership | 0.031 | -0.194 | -0.352*** | 0.118** | -0.271** |
|  | (0.046) | (0.124) | (0.097) | (0.059) | (0.126) |
| Livestock ownership (in TLU) | 0.004 | -0.009** | -0.004 | -0.002 | -0.003 |
|  | (0.003) | (0.004) | (0.004) | (0.003) | (0.005) |
| Kebele fixed effect | Yes | Yes | Yes | Yes | Yes |
| Mean for control group | 85.6 | 82.6 | 28.7 | 89.8 | 45.5 |
| Observations | 490 | 490 | 490 | 490 | 490 |
| R-squared | 0.146 | 0.238 | 0.346 | 0.136 | 0.255 |

Source: Authors’ calculation based on data from the 2014 wheat growers’ survey. Robust standard errors in parentheses. *** p<0.01, ** p<0.05, * p<0.1

The following tables replicate Tables 6-9, with no covariates included, to demonstrate that coefficient estimates of interest are not qualitatively different.

Appendix Table A.7: Farmers knowledge of the promotional wheat package

|  | Information on ATA Wheat initiative *(%, yes)* | Training on wheat production method *(%, yes)* | Package include certified seed *(%, yes)* | Package include reduced seed rate *(%, yes)* | Package include row planting *(%, yes)* | Urea application rate *(kg/ha)* | DAP application rate *(kg/ha)* |
| --- | --- | --- | --- | --- | --- | --- | --- |
| Variables |  |  |  |  |  |  |  |
|  |  |  |  |  |  |  |  |
| Full package | 0.375*** | 0.542*** | 0.0201 | 0.0414 | -0.000532 | 3.883 | -3.447 |
|  | (0.0431) | (0.0423) | (0.0134) | (0.0347) | (0.0324) | (5.262) | (6.100) |
| Marketing assistance | -0.00892 | 0.0900 | 0.0184 | -0.0311 | -0.0762** | 3.205 | -3.651 |
|  | (0.0598) | (0.0569) | (0.0158) | (0.0457) | (0.0381) | (6.773) | (6.881) |
| Constant | 0.567*** | 0.378*** | 0.974*** | 0.846*** | 0.855*** | 119.9*** | 144.0*** |
|  | (0.0382) | (0.0371) | (0.0128) | (0.0273) | (0.0246) | (3.539) | (4.629) |
|  |  |  |  |  |  |  |  |
| Observations | 469 | 469 | 451 | 469 | 469 | 428 | 430 |
| R-squared | 0.237 | 0.371 | 0.074 | 0.158 | 0.400 | 0.530 | 0.569 |

Robust standard errors in parentheses

*** p<0.01, ** p<0.05, * p<0.1

Appendix Table A.8: Farmers experiences with the services provided under the Wheat Initiative

|  | Received certified seed *(%, yes on time)* | Quality of seed *(%, very good)* | Received Urea for free *(%, yes on time)* | Quality of Urea *(%, very good)* | Received gypsum for free *(%, yes on time)* | Received marketing assistance *(%, yes)* | Grow wheat differently in 2013 meher *(%, yes)* | Know a friend/neighbor grow wheat differently in 2013 meher *(%, yes)* |
| --- | --- | --- | --- | --- | --- | --- | --- | --- |
| Variables |  |  |  |  |  |  |  |  |
|  |  |  |  |  |  |  |  |  |
| Full package | 0.644*** | 0.481*** | 0.675*** | 0.475*** | 0.300*** | 0.0336 | 0.381*** | 0.0462 |
|  | (0.0375) | (0.0441) | (0.0394) | (0.0398) | (0.0324) | (0.0350) | (0.0431) | (0.0409) |
| Marketing assistance | -0.0444 | 0.0173 | -0.00255 | 0.00848 | -0.00741 | -0.00784 | 0.0470 | -0.0932* |
|  | (0.0466) | (0.0463) | (0.0455) | (0.0343) | (0.0323) | (0.0391) | (0.0563) | (0.0492) |
| Constant | 0.291*** | 0.206*** | 0.168*** | 0.0742*** | 0.0448** | 0.131*** | 0.494*** | 0.767*** |
|  | (0.0302) | (0.0287) | (0.0289) | (0.0212) | (0.0208) | (0.0252) | (0.0359) | (0.0303) |
|  |  |  |  |  |  |  |  |  |
| Observations | 490 | 490 | 490 | 490 | 490 | 490 | 490 | 490 |
| R-squared | 0.524 | 0.320 | 0.480 | 0.350 | 0.388 | 0.170 | 0.273 | 0.199 |

Robust standard errors in parentheses

*** p<0.01, ** p<0.05, * p<0.1

Appendix Table A.9: Farmers implementation of the promotional wheat package

|  | Certified seed *(%, yes)* | Certified seed quantity *(kg/ha)* | Urea *(%, yes)* | Urea applied *(kg/ha)* | DAP *(%, yes)* | DAP applied *(kg/ha)* | Gypsum *(%, yes)* | Pesticide *(%, yes)* | Herbicide *(%, yes)* | Row planting *(%, yes)* |
| --- | --- | --- | --- | --- | --- | --- | --- | --- | --- | --- |
| Variables |  |  |  |  |  |  |  |  |  |  |
|  |  |  |  |  |  |  |  |  |  |  |
| Full package | 0.467*** | -24.30*** | 0.0982*** | 25.99*** | -0.00417 | -0.109 | 0.290*** | 0.0344 | -0.0360 | 0.324*** |
|  | (0.0388) | (9.031) | (0.0221) | (8.944) | (0.00524) | (8.181) | (0.0320) | (0.0305) | (0.0299) | (0.0397) |
| Marketing assistance | 0.0160 | -11.83 | 0.000751 | -3.120 | -0.00741 | -8.942 | 0.00110 | 0.0332 | -0.00246 | 0.00760 |
|  | (0.0535) | (10.69) | (0.0292) | (8.494) | (0.00802) | (8.698) | (0.0330) | (0.0341) | (0.0375) | (0.0459) |
| Constant | 0.512*** | 177.9*** | 0.905*** | 128.1*** | 1.000*** | 154.4*** | 0.0442** | 0.100*** | 0.603*** | 0.274*** |
|  | (0.0349) | (7.594) | (0.0196) | (5.558) | (0.00138) | (5.497) | (0.0210) | (0.0215) | (0.0235) | (0.0307) |
|  |  |  |  |  |  |  |  |  |  |  |
| Observations | 490 | 346 | 490 | 464 | 490 | 488 | 490 | 490 | 490 | 490 |
| R-squared | 0.374 | 0.285 | 0.247 | 0.455 | 0.072 | 0.483 | 0.391 | 0.248 | 0.671 | 0.483 |

Robust standard errors in parentheses

*** p<0.01, ** p<0.05, * p<0.1

Appendix Table A.10: Farmers plans for adopting the promotional wheat package in the following season (2014 meher season)

|  | Plan to buy/apply . . . | | | | |
| --- | --- | --- | --- | --- | --- |
|  | Seed if on cash *(%, yes)* | Seed if on credit *(%, yes)* | Row planting | Reduced seeding rate | Recommended (more) fertilizer |
| Variables |  |  |  |  |  |
|  |  |  |  |  |  |
| Full package | -0.0337 | -0.115*** | 0.122*** | 0.0515* | 0.0456 |
|  | (0.0374) | (0.0431) | (0.0442) | (0.0282) | (0.0495) |
| Marketing assistance | -0.0273 | -0.0789 | 0.0745 | 0.0104 | 0.0231 |
|  | (0.0427) | (0.0481) | (0.0485) | (0.0354) | (0.0550) |
| Constant | 0.859*** | 0.819*** | 0.279*** | 0.897*** | 0.451*** |
|  | (0.0262) | (0.0291) | (0.0330) | (0.0238) | (0.0371) |
|  |  |  |  |  |  |
| Observations | 490 | 490 | 490 | 490 | 490 |
| R-squared | 0.117 | 0.141 | 0.308 | 0.069 | 0.202 |

Robust standard errors in parentheses

*** p<0.01, ** p<0.05, * p<0.1

The following Table tests whether full package farmers or market assistance farmers have differential rates of attrition from the crop cut; we find no differences by groups.

Appendix Table A.11. Characterizing non-responses in crop cut production

|  | Dependent variable: Crop-cuts (1=Yes) | |
| --- | --- | --- |
| Variables | (1) | (2) |
|  |  |  |
| Full package | 0.010 | 0.071 |
|  | (0.031) | (0.049) |
| Marketing assistance | 0.011 | 0.010 |
|  | (0.034) | (0.045) |
| Age of HH head |  | -0.003 |
|  |  | (0.003) |
| Gender of HH head |  | -0.041 |
|  |  | (0.047) |
| Household size |  | -0.009 |
|  |  | (0.014) |
| Education of HH head |  | -0.110* |
|  |  | (0.057) |
| Landholding size |  | 0.029*** |
|  |  | (0.011) |
| Soil fertility |  |  |
| Medium |  | -0.017 |
|  |  | (0.045) |
| Poor |  | 0.100 |
|  |  | (0.073) |
| Soil color |  |  |
| Black |  | -0.065 |
|  |  | (0.064) |
| Grey or sandy |  | -0.023 |
|  |  | (0.057) |
| Plot distance from dwelling |  | -0.000 |
|  |  | (0.002) |
| Own plot (1=Yes) |  | 0.006 |
|  |  | (0.061) |
| Seeding rate (kg/ha) |  | 0.001 |
|  |  | (0.000) |
| Urea fertilizer (kg/ha) |  | 0.000 |
|  |  | (0.001) |
| DAP fertilizer (kg/ha) |  | -0.001** |
|  |  | (0.001) |
| Row planting (1=Yes) |  | -0.233*** |
|  |  | (0.080) |
| Constant | 0.741*** | 1.188*** |
|  | (0.064) | (0.182) |
| Observations | 488 | 485 |

Note: Robust standard errors clustered at the kebele level in parentheses. *** p<0.01, ** p<0.05, * p<0.10.
