# Supplementary material for: The impact of the use of new technologies on farmers’ wheat yield in Ethiopia: evidence from a randomized control trial
Source: Agric Econ. 2018 Jul 2;49(4):409–21. doi: 10.1111/agec.12425 (PMC6108534; doi:10.1111/agec.12425)
Supplement: Supplementary file 2 — Supporting Information [file AGEC-49-409-s002.pdf]

Appendix

Appendix Table A.1. Average Yields by Crop-Cut Status

	Sample farmers with crop-cut data (n=367)		Sample farmers without crop-cut data (n=122)	
	Mean	Std. Error	Mean	Std. Error
Predicted yield	3.11***	0.08	4.37	0.16
Recall yield	2.73***	0.08	4.06	0.18

Appendix Table A.2.: Power Calculations for Realized Differences in Averages

Outcome	Average, Treatment	Average, Control	Std. Deviation, Treatment	Std. Deviation, Control	Number of Obs., treatment	Number of Obs. control	Power to detect difference at 5 percent level
Panel A (full package vs control)							
Yield: crop-cut	7.802	7.735	0.6645	0.6546	124	148	0.132
Yield: predicted	8.069	7.917	0.5780	0.6308	194	163	0.653
Yield: recall	7.869	7.779	0.6940	0.6817	197	166	0.238
Panel B (market vs control)							
Yield: crop-cut	7.785	7.735	0.5854	0.6546	95	148	0.095
Yield: predicted	7.943	7.917	0.5806	0.6308	125	163	0.066
Yield: recall	7.828	7.779	0.7221	0.6817	126	166	0.091

Appendix Table A.3. Farmers' knowledge of the promotional wheat package

Variables	Information on ATA Wheat initiative *(%, yes)*	Training on wheat production method *(%, yes)*	Package include certified seed *(%, yes)*	Package include reduced seed rate *(%, yes)*	Package include row planting *(%, yes)*	Urea application rate *(kg/ha)*	DAP application rate *(kg/ha)*
Full package	0.382***	0.589***	0.011	0.062	0.002	-1.679	-4.682
	(0.051)	(0.052)	(0.018)	(0.045)	(0.043)	(8.148)	(8.987)
Marketing assistance	-0.007	0.106*	0.023	-0.027	-0.074*	3.600	-1.863
	(0.061)	(0.056)	(0.016)	(0.047)	(0.039)	(6.953)	(7.140)
Model farmer	0.061	0.228***	0.017*	0.091*	0.053	-5.776	-4.631
	(0.069)	(0.070)	(0.010)	(0.048)	(0.037)	(9.835)	(8.128)
Female farmer	-0.004	0.034	-0.078	-0.066	-0.052	2.014	1.857
	(0.100)	(0.085)	(0.058)	(0.098)	(0.084)	(8.280)	(9.231)
Treatment × model	-0.094	-0.165**	-0.004	-0.047	-0.031	19.409	4.839
	(0.083)	(0.083)	(0.015)	(0.067)	(0.060)	(12.095)	(11.556)
Treatment × female	0.065	-0.059	0.089	-0.003	0.050	0.865	5.479
	(0.108)	(0.102)	(0.058)	(0.117)	(0.099)	(11.332)	(13.103)
Age of household head	-0.002	0.003	-0.000	0.001	0.000	0.048	-0.101
	(0.002)	(0.002)	(0.001)	(0.002)	(0.002)	(0.298)	(0.318)
Education of household head	-0.006	0.017	-0.011	0.017	0.014	3.639	2.996
	(0.026)	(0.025)	(0.010)	(0.025)	(0.018)	(3.526)	(4.138)
Landholding size	-0.004	-0.009	-0.001	-0.014	0.009	-1.254	-1.933
	(0.015)	(0.012)	(0.002)	(0.010)	(0.009)	(1.397)	(1.407)
Household size	0.010	0.006	-0.000	-0.004	0.000	0.562	1.244
	(0.010)	(0.009)	(0.003)	(0.008)	(0.007)	(1.053)	(1.157)
Black soil	0.031	-0.115**	0.003	-0.063	0.002	-2.663	-8.778
	(0.057)	(0.055)	(0.018)	(0.054)	(0.043)	(7.543)	(6.939)
Gray/sandy soil	-0.005	-0.170**	0.016	-0.024	0.005	-4.135	-1.837
	(0.067)	(0.066)	(0.016)	(0.054)	(0.048)	(8.536)	(9.056)
Distance to plot	-0.001	0.000	0.000	0.001	-0.002	0.335	0.181
	(0.002)	(0.001)	(0.000)	(0.002)	(0.001)	(0.233)	(0.262)
Radio ownership	0.109**	0.015	0.014	-0.032	0.028	3.415	1.093
	(0.051)	(0.047)	(0.018)	(0.043)	(0.040)	(6.486)	(7.435)
Television ownership	-0.082	-0.050	-0.020	-0.016	-0.027	-12.186*	-20.421**
	(0.066)	(0.060)	(0.023)	(0.047)	(0.044)	(7.336)	(8.933)
Cellphone ownership	0.021	0.017	-0.000	-0.058	-0.037	-10.144	-5.838
	(0.061)	(0.057)	(0.023)	(0.049)	(0.045)	(8.994)	(8.357)
Bicycle ownership	0.018	0.194*	0.001	-0.030	-0.089	8.751	18.194
	(0.113)	(0.106)	(0.013)	(0.063)	(0.086)	(20.331)	(18.649)
Car ownership	-0.059	-0.280**	-0.004	-0.037	-0.020	15.545	3.228
	(0.111)	(0.114)	(0.012)	(0.060)	(0.062)	(9.639)	(14.651)
Livestock ownership (in TLU)	0.001	-0.001	0.000	0.002	0.000	0.015	0.615
	(0.003)	(0.003)	(0.001)	(0.002)	(0.002)	(0.357)	(0.473)
Constant	0.524***	0.201	0.993***	0.891***	0.829***	111.534***	138.363***
	(0.168)	(0.148)	(0.052)	(0.142)	(0.113)	(23.322)	(27.033)
Kebele fixed effect	Yes	Yes	Yes	Yes	Yes	Yes	Yes
Mean for control group	57.4	38.3	94.4	87.9	91.1	163.5	185.3

Observations	469	469	451	469	469	428	430
R-squared	0.260	0.425	0.112	0.185	0.416	0.544	0.584

Source: Authors' calculation based on data from the 2014 wheat growers' survey. Robust standard errors in parentheses. *** p<0.01, ** p<0.05, * p<0.1.

Appendix Table A.4. Farmers' experiences with the services provided under the Wheat Initiative

Variables	Received certified seed *(%, yes on time)*	Quality of seed *(%, very good)*	Received Urea for free *(%, yes on time)*	Quality of Urea *(%, very good)*	Received gypsum for free *(%, yes on time)*	Received marketing assistance *(%, yes)*	Grow wheat differently in 2013 meher *(%, yes)*	Know a friend/neighbor grow wheat differently in 2013 meher *(%, yes)*
Full package	0.664***	0.460***	0.750***	0.480***	0.291***	0.037	0.325***	0.040
	(0.046)	(0.061)	(0.051)	(0.056)	(0.043)	(0.047)	(0.057)	(0.056)
Marketing assistance	-0.051	0.025	-0.003	0.017	-0.002	-0.004	0.042	-0.089*
	(0.047)	(0.048)	(0.045)	(0.037)	(0.034)	(0.040)	(0.057)	(0.050)
Model farmer	0.054	0.066	0.055	0.043	0.014	0.064	0.040	-0.041
	(0.055)	(0.059)	(0.054)	(0.044)	(0.041)	(0.049)	(0.066)	(0.058)
Female farmer	0.132	-0.004	0.201**	0.020	-0.059	-0.062	0.039	0.115
	(0.097)	(0.086)	(0.095)	(0.061)	(0.056)	(0.073)	(0.104)	(0.079)
Treatment × model	-0.062	-0.041	-0.111	0.014	0.013	-0.039	0.055	0.055
	(0.074)	(0.093)	(0.080)	(0.084)	(0.065)	(0.074)	(0.083)	(0.082)
Treatment × female	-0.120	0.118	-0.276**	-0.032	0.053	0.070	0.044	-0.106
	(0.107)	(0.121)	(0.118)	(0.108)	(0.084)	(0.097)	(0.117)	(0.103)
Age of household head	0.001	0.004	0.002	0.004*	-0.000	-0.000	-0.002	0.002
	(0.002)	(0.002)	(0.002)	(0.002)	(0.002)	(0.002)	(0.002)	(0.002)
Education of household head	0.020	0.006	0.011	0.006	0.002	-0.033*	0.003	0.008
	(0.022)	(0.024)	(0.024)	(0.020)	(0.018)	(0.019)	(0.026)	(0.026)
Landholding size	-0.009	-0.019	-0.006	-0.019	0.023**	0.009	0.011	0.018
	(0.012)	(0.014)	(0.013)	(0.012)	(0.010)	(0.010)	(0.013)	(0.013)
Household size	-0.006	-0.006	-0.003	0.002	0.002	0.005	-0.003	-0.002
	(0.009)	(0.011)	(0.009)	(0.009)	(0.007)	(0.008)	(0.010)	(0.009)
Black soil	0.015	0.029	0.027	-0.012	0.032	-0.023	-0.021	-0.029
	(0.051)	(0.059)	(0.053)	(0.051)	(0.044)	(0.047)	(0.058)	(0.060)
Gray/sandy soil	-0.061	-0.032	0.028	-0.014	0.062	0.054	-0.061	-0.042
	(0.055)	(0.064)	(0.059)	(0.056)	(0.050)	(0.050)	(0.066)	(0.065)
Distance to plot	0.000	-0.002	-0.001	0.000	-0.001	-0.000	0.001	-0.000
	(0.001)	(0.001)	(0.001)	(0.001)	(0.001)	(0.001)	(0.001)	(0.002)
Radio ownership	0.005	0.036	0.017	0.033	-0.001	0.063	-0.097*	0.106**
	(0.043)	(0.050)	(0.041)	(0.042)	(0.036)	(0.039)	(0.050)	(0.049)
Television ownership	0.029	0.004	-0.041	0.070	-0.085*	0.069	0.009	-0.011
	(0.056)	(0.065)	(0.059)	(0.058)	(0.043)	(0.062)	(0.069)	(0.060)
Cellphone ownership	-0.040	-0.044	-0.115**	-0.074	0.024	0.025	0.105*	0.015
	(0.049)	(0.055)	(0.050)	(0.046)	(0.042)	(0.045)	(0.058)	(0.055)
Bicycle ownership	-0.030	-0.025	0.080	-0.069	0.096	-0.088	-0.265***	-0.185
	(0.114)	(0.114)	(0.104)	(0.093)	(0.076)	(0.074)	(0.099)	(0.122)
Car ownership	-0.111	-0.097	0.023	-0.024	-0.056	-0.145	-0.104	0.035
	(0.079)	(0.094)	(0.092)	(0.090)	(0.067)	(0.111)	(0.099)	(0.077)
Livestock ownership (in TLU)	-0.000	0.002	0.000	-0.002	-0.003	-0.004	0.004	-0.001
	(0.003)	(0.004)	(0.003)	(0.004)	(0.003)	(0.003)	(0.003)	(0.004)

Kebele fixed effect	Yes	Yes	Yes	Yes	Yes	Yes	Yes	Yes
Mean for control group	29.3	20.9	16.7	32.1	4.7	13.1	49.1	76.6
Observations	490	490	490	490	490	490	490	490
R-squared	0.537	0.336	0.500	0.369	0.408	0.203	0.306	0.226

Source: Authors' calculation based on data from the 2014 wheat growers' survey. Robust standard errors in parentheses. *** p<0.01, ** p<0.05, * p<0.1

Appendix Table A.5. Farmers' implementation of the promotional wheat package

Variables	Certified seed *(%, yes)*	Certified seed quantity *(kg/ha)*	Urea *(%, yes)*	Urea applied *(kg/ha)*	DAP *(%, yes)*	DAP applied *(kg/ha)*	Gypsum *(%, yes)*	Pesticide *(%, yes)*	Herbicide *(%, yes)*	Row planting *(%, yes)*
Full package	0.490***	-22.783*	0.113***	15.774	-0.002	-3.910	0.281***	0.006	-0.036	0.367***
	(0.048)	(11.726)	(0.028)	(11.905)	(0.003)	(9.867)	(0.043)	(0.041)	(0.036)	(0.051)
Marketing assistance	0.009	-7.885	0.003	-4.306	-0.009	-11.249	0.009	0.027	-0.010	0.003
	(0.054)	(10.504)	(0.028)	(8.770)	(0.009)	(8.737)	(0.034)	(0.036)	(0.038)	(0.047)
Model farmer	0.105*	-15.719	0.031	-1.432	-0.015	6.824	0.013	-0.001	0.022	0.060
	(0.064)	(11.726)	(0.031)	(9.407)	(0.014)	(10.269)	(0.042)	(0.043)	(0.050)	(0.056)
Female farmer	0.109	-36.069*	0.034	-2.844	-0.001	-5.872	-0.062	-0.038	0.116*	0.005
	(0.095)	(19.039)	(0.037)	(15.804)	(0.004)	(12.227)	(0.057)	(0.032)	(0.067)	(0.075)
Treatment × model	-0.122*	-2.094	-0.056	5.820	-0.002	-7.685	0.024	0.024	-0.022	-0.102
	(0.073)	(14.898)	(0.040)	(17.033)	(0.016)	(16.303)	(0.065)	(0.062)	(0.066)	(0.075)
Treatment × female	-0.088	27.171	-0.039	18.847	0.004	11.746	0.047	0.095	-0.073	-0.037
	(0.101)	(19.285)	(0.043)	(21.907)	(0.007)	(17.414)	(0.082)	(0.066)	(0.080)	(0.093)
Age of household head	0.001	-0.250	0.000	-0.165	-0.000	-0.173	0.000	-0.001	-0.002	-0.003
	(0.002)	(0.409)	(0.001)	(0.367)	(0.000)	(0.373)	(0.002)	(0.002)	(0.001)	(0.002)
Education of household head	0.013	-8.699*	0.011	-3.392	0.000	2.230	0.000	-0.000	-0.007	0.001
	(0.023)	(5.100)	(0.013)	(4.558)	(0.004)	(4.176)	(0.017)	(0.018)	(0.017)	(0.023)
Landholding size	0.003	-6.946***	0.007	-4.110**	0.003	-5.701***	0.018*	0.012	-0.001	0.012
	(0.014)	(2.329)	(0.007)	(1.887)	(0.003)	(2.161)	(0.010)	(0.014)	(0.008)	(0.013)
Household size	-0.017*	-0.932	-0.007	-3.450**	-0.000	-2.168	0.000	-0.002	0.005	0.005
	(0.009)	(1.767)	(0.006)	(1.730)	(0.001)	(1.532)	(0.007)	(0.008)	(0.006)	(0.009)
Black soil	-0.092*	0.485	0.012	13.419	0.005	8.665	-0.006	-0.031	0.021	-0.078
	(0.049)	(10.126)	(0.026)	(11.174)	(0.006)	(10.619)	(0.045)	(0.037)	(0.042)	(0.049)
Gray/sandy soil	-0.106*	17.485	0.013	2.245	0.011	15.259	0.025	-0.055	0.035	0.011
	(0.057)	(13.245)	(0.032)	(13.004)	(0.008)	(13.615)	(0.049)	(0.042)	(0.049)	(0.059)
Distance to plot	0.000	-0.252	-0.000	-0.632**	0.000	-0.560**	-0.001	0.000	-0.000	0.001
	(0.001)	(0.360)	(0.001)	(0.302)	(0.000)	(0.272)	(0.001)	(0.001)	(0.001)	(0.001)
Radio ownership	-0.057	8.159	-0.027	-11.285	-0.008	0.444	0.005	0.007	-0.010	0.096**
	(0.042)	(9.236)	(0.022)	(9.563)	(0.008)	(8.455)	(0.036)	(0.036)	(0.035)	(0.042)
Television ownership	-0.011	23.813**	-0.072*	-9.134	0.008	2.894	-0.069	0.007	-0.014	-0.013
	(0.063)	(10.614)	(0.042)	(10.466)	(0.009)	(13.827)	(0.045)	(0.053)	(0.046)	(0.060)
Cellphone ownership	0.063	-20.877*	-0.005	3.403	0.007	2.656	0.017	0.043	0.052	0.001
	(0.050)	(12.098)	(0.025)	(12.055)	(0.008)	(10.003)	(0.042)	(0.031)	(0.037)	(0.047)
Bicycle ownership	0.100	-12.238	-0.003	21.689	0.012	14.967	0.096	-0.048	0.079	-0.051
	(0.119)	(14.920)	(0.098)	(17.605)	(0.011)	(17.260)	(0.075)	(0.090)	(0.077)	(0.105)
Car ownership	-0.141	-9.889	-0.171	-0.871	0.001	0.752	-0.061	0.096	0.025	-0.044
	(0.108)	(17.079)	(0.106)	(12.691)	(0.009)	(15.715)	(0.066)	(0.134)	(0.043)	(0.116)
Livestock ownership (in TLU)	0.006**	1.740***	0.003	1.237**	-0.000	1.623***	-0.002	0.000	-0.000	-0.002

	(0.003)	(0.601)	(0.002)	(0.535)	(0.000)	(0.592)	(0.003)	(0.003)	(0.002)	(0.004)
Kebele fixed effect	Yes	Yes	Yes	Yes	Yes	Yes	Yes	Yes	Yes	Yes
Mean for control group	51.5	177.9	91.0	129.6	100.0	153.0	4.7	8.9	59.8	26.9
Observations	490	346	490	464	490	488	490	490	490	490
R-squared	0.398	0.358	0.288	0.479	0.097	0.502	0.407	0.262	0.680	0.502

Source: Authors' calculation based on data from the 2014 wheat growers' survey. Robust standard errors in parentheses. *** p<0.01, ** p<0.05, * p<0.1

Appendix Table A.6. Farmers' plans for adopting the promotional wheat package in the following season (2014 *meher* season)

	Plan to buy/apply . . .				
Variables	Seed if on cash *(%, yes)*	Seed if on credit *(%, yes)*	Row planting	Reduced seeding rate	Recommended (more) fertilizer
Full package	-0.016	-0.075	0.184***	0.074**	0.102
	(0.047)	(0.056)	(0.055)	(0.030)	(0.064)
Marketing assistance	-0.038	-0.064	0.081	0.008	0.023
	(0.043)	(0.047)	(0.050)	(0.034)	(0.056)
Model farmer	-0.005	-0.110*	0.004	0.028	-0.050
	(0.050)	(0.060)	(0.060)	(0.040)	(0.069)
Female farmer	-0.065	-0.024	0.069	-0.004	-0.066
	(0.090)	(0.082)	(0.084)	(0.068)	(0.086)
Treatment × model	-0.060	-0.038	-0.110	-0.072	-0.054
	(0.076)	(0.092)	(0.086)	(0.053)	(0.094)
Treatment × female	0.057	0.074	-0.142	0.007	-0.063
	(0.114)	(0.107)	(0.112)	(0.076)	(0.118)
Age of household head	-0.001	-0.004*	-0.003	-0.004***	-0.006**
	(0.002)	(0.002)	(0.002)	(0.002)	(0.002)
Education of household head	0.044*	0.016	0.013	0.011	-0.040
	(0.023)	(0.023)	(0.023)	(0.017)	(0.027)
Landholding size	-0.004	-0.004	0.004	0.007	0.013
	(0.010)	(0.013)	(0.014)	(0.008)	(0.017)
Household size	-0.001	0.025***	0.013	0.017***	0.028**
	(0.008)	(0.010)	(0.010)	(0.007)	(0.011)
Black soil	0.004	-0.130**	0.042	-0.026	0.068
	(0.053)	(0.051)	(0.053)	(0.031)	(0.058)
Gray/sandy soil	0.068	-0.144**	0.074	-0.056	-0.036
	(0.055)	(0.060)	(0.063)	(0.038)	(0.068)
Distance to plot	-0.000	0.001	-0.001	-0.000	0.002
	(0.001)	(0.002)	(0.001)	(0.001)	(0.002)
Radio ownership	0.015	-0.017	-0.017	0.008	0.010
	(0.043)	(0.044)	(0.050)	(0.033)	(0.055)
Television ownership	0.047	0.031	-0.036	-0.056	0.034
	(0.055)	(0.066)	(0.075)	(0.052)	(0.079)
Cellphone ownership	-0.023	0.002	0.024	0.073*	-0.053
	(0.050)	(0.053)	(0.056)	(0.039)	(0.059)
Bicycle ownership	-0.063	0.017	0.023	0.047	0.258**
	(0.083)	(0.111)	(0.126)	(0.063)	(0.118)

Car ownership	0.031	-0.194	-0.352***	0.118**	-0.271**
	(0.046)	(0.124)	(0.097)	(0.059)	(0.126)
Livestock ownership (in TLU)	0.004	-0.009**	-0.004	-0.002	-0.003
	(0.003)	(0.004)	(0.004)	(0.003)	(0.005)
Kebele fixed effect	Yes	Yes	Yes	Yes	Yes
Mean for control group	85.6	82.6	28.7	89.8	45.5
Observations	490	490	490	490	490
R-squared	0.146	0.238	0.346	0.136	0.255

Source: Authors' calculation based on data from the 2014 wheat growers' survey. Robust standard errors in parentheses. *** p<0.01, ** p<0.05, * p<0.1

The following tables replicate Tables 6-9, with no covariates included, to demonstrate that coefficient estimates of interest are not qualitatively different.

Appendix Table A.7: Farmers knowledge of the promotional wheat package

Variables	Information on ATA Wheat initiative *(%, yes)*	Training on wheat production method *(%, yes)*	Package include certified seed *(%, yes)*	Package include reduced seed rate *(%, yes)*	Package include row planting *(%, yes)*	Urea application rate *(kg/ha)*	DAP application rate *(kg/ha)*
Full package	0.375***	0.542***	0.0201	0.0414	-0.000532	3.883	-3.447
	(0.0431)	(0.0423)	(0.0134)	(0.0347)	(0.0324)	(5.262)	(6.100)
Marketing assistance	-0.00892	0.0900	0.0184	-0.0311	-0.0762**	3.205	-3.651
	(0.0598)	(0.0569)	(0.0158)	(0.0457)	(0.0381)	(6.773)	(6.881)
Constant	0.567***	0.378***	0.974***	0.846***	0.855***	119.9***	144.0***
	(0.0382)	(0.0371)	(0.0128)	(0.0273)	(0.0246)	(3.539)	(4.629)
Observations	469	469	451	469	469	428	430
R-squared	0.237	0.371	0.074	0.158	0.400	0.530	0.569

Robust standard errors in parentheses
*** p<0.01, ** p<0.05, * p<0.1

Appendix Table A.8: Farmers experiences with the services provided under the Wheat Initiative

Variables	Received certified seed *(%, yes on time)*	Quality of seed *(%, very good)*	Received Urea for free *(%, yes on time)*	Quality of Urea *(%, very good)*	Received gypsum for free *(%, yes on time)*	Received marketing assistance *(%, yes)*	Grow wheat differently in 2013 meher *(%, yes)*	Know a friend/neighbor grow wheat differently in 2013 meher *(%, yes)*
Full package	0.644***	0.481***	0.675***	0.475***	0.300***	0.0336	0.381***	0.0462
	(0.0375)	(0.0441)	(0.0394)	(0.0398)	(0.0324)	(0.0350)	(0.0431)	(0.0409)
Marketing assistance	-0.0444	0.0173	-0.00255	0.00848	-0.00741	-0.00784	0.0470	-0.0932*
	(0.0466)	(0.0463)	(0.0455)	(0.0343)	(0.0323)	(0.0391)	(0.0563)	(0.0492)
Constant	0.291***	0.206***	0.168***	0.0742***	0.0448**	0.131***	0.494***	0.767***
	(0.0302)	(0.0287)	(0.0289)	(0.0212)	(0.0208)	(0.0252)	(0.0359)	(0.0303)
Observations	490	490	490	490	490	490	490	490
R-squared	0.524	0.320	0.480	0.350	0.388	0.170	0.273	0.199

Robust standard errors in parentheses

*** p<0.01, ** p<0.05, * p<0.1

Appendix Table A.9: Farmers implementation of the promotional wheat package

Variables	Certified seed *(%, yes)*	Certified seed quantity *(kg/ha)*	Urea *(%, yes)*	Urea applied *(kg/ha)*	DAP *(%, yes)*	DAP applied *(kg/ha)*	Gypsum *(%, yes)*	Pesticide *(%, yes)*	Herbicide *(%, yes)*	Row planting *(%, yes)*
Full package	0.467***	-24.30***	0.0982***	25.99***	-0.00417	-0.109	0.290***	0.0344	-0.0360	0.324***
	(0.0388)	(9.031)	(0.0221)	(8.944)	(0.00524)	(8.181)	(0.0320)	(0.0305)	(0.0299)	(0.0397)
Marketing assistance	0.0160	-11.83	0.000751	-3.120	-0.00741	-8.942	0.00110	0.0332	-0.00246	0.00760
	(0.0535)	(10.69)	(0.0292)	(8.494)	(0.00802)	(8.698)	(0.0330)	(0.0341)	(0.0375)	(0.0459)
Constant	0.512***	177.9***	0.905***	128.1***	1.000***	154.4***	0.0442**	0.100***	0.603***	0.274***
	(0.0349)	(7.594)	(0.0196)	(5.558)	(0.00138)	(5.497)	(0.0210)	(0.0215)	(0.0235)	(0.0307)
Observations	490	346	490	464	490	488	490	490	490	490
R-squared	0.374	0.285	0.247	0.455	0.072	0.483	0.391	0.248	0.671	0.483

Robust standard errors in parentheses
*** p<0.01, ** p<0.05, * p<0.1

Appendix Table A.10: Farmers plans for adopting the promotional wheat package in the following season (2014 meher season)

	Plan to buy/apply . . .				
Variables	Seed if on cash *(%, yes)*	Seed if on credit *(%, yes)*	Row planting	Reduced seeding rate	Recommended (more) fertilizer
Full package	-0.0337	-0.115***	0.122***	0.0515*	0.0456
	(0.0374)	(0.0431)	(0.0442)	(0.0282)	(0.0495)
Marketing assistance	-0.0273	-0.0789	0.0745	0.0104	0.0231
	(0.0427)	(0.0481)	(0.0485)	(0.0354)	(0.0550)
Constant	0.859***	0.819***	0.279***	0.897***	0.451***
	(0.0262)	(0.0291)	(0.0330)	(0.0238)	(0.0371)
Observations	490	490	490	490	490
R-squared	0.117	0.141	0.308	0.069	0.202

Robust standard errors in parentheses
*** p<0.01, ** p<0.05, * p<0.1

The following Table tests whether full package farmers or market assistance farmers have differential rates of attrition from the crop cut; we find no differences by groups.

Appendix Table A.11. Characterizing non-responses in crop cut production

	Dependent variable: Crop-cuts (1=Yes)	
Variables	(1)	(2)
Full package	0.010	0.071
	(0.031)	(0.049)
Marketing assistance	0.011	0.010
	(0.034)	(0.045)
Age of HH head		-0.003
		(0.003)
Gender of HH head		-0.041
		(0.047)
Household size		-0.009
		(0.014)
Education of HH head		-0.110*
		(0.057)
Landholding size		0.029***
		(0.011)
Soil fertility		
Medium		-0.017
		(0.045)
Poor		0.100
		(0.073)
Soil color		
Black		-0.065
		(0.064)
Grey or sandy		-0.023
		(0.057)
Plot distance from dwelling		-0.000
		(0.002)
Own plot (1=Yes)		0.006
		(0.061)
Seeding rate (kg/ha)		0.001
		(0.000)
Urea fertilizer (kg/ha)		0.000
		(0.001)
DAP fertilizer (kg/ha)		-0.001**
		(0.001)
Row planting (1=Yes)		-0.233***
		(0.080)
Constant	0.741***	1.188***
	(0.064)	(0.182)
Observations	488	485

Note: Robust standard errors clustered at the kebele level in parentheses. *** p<0.01, ** p<0.05, * p<0.10.
